# Functional Characterization of EngA^MS^, a P-Loop GTPase of *Mycobacterium smegmatis*

**DOI:** 10.1371/journal.pone.0034571

**Published:** 2012-04-10

**Authors:** Nisheeth Agarwal, Madhu Pareek, Preeti Thakur, Vibha Pathak

**Affiliations:** Vaccine and Infectious Disease Research Center, Translational Health Science and Technology Institute, Gurgaon, Haryana, India; The University of Hong Kong, China

## Abstract

Bacterial P-loop GTPases belong to a family of proteins that selectively hydrolyze a small molecule guanosine tri-phosphate (GTP) to guanosine di-phosphate (GDP) and inorganic phosphate, and regulate several essential cellular activities such as cell division, chromosomal segregation and ribosomal assembly. A comparative genome sequence analysis of different mycobacterial species indicates the presence of multiple P-loop GTPases that exhibit highly conserved motifs. However, an exact function of most of these GTPases in mycobacteria remains elusive. In the present study we characterized the function of a P-loop GTPase in mycobacteria by employing an EngA homologue from *Mycobacterium smegmatis*, encoded by an open reading frame, designated as *MSMEG_3738*. Amino acid sequence alignment and phylogenetic analysis suggest that MSMEG_3738 (termed as EngA^MS^) is highly conserved in mycobacteria. Homology modeling of EngA^MS^ reveals a cloverleaf structure comprising of α/β fold typical to EngA family of GTPases. Recombinant EngA^MS^ purified from *E. coli* exhibits a GTP hydrolysis activity which is inhibited by the presence of GDP. Interestingly, the EngA^MS^ protein is co-eluted with 16S and 23S ribosomal RNA during purification and exhibits association with 30S, 50S and 70S ribosomal subunits. Further studies demonstrate that GTP is essential for interaction of EngA^MS^ with 50S subunit of ribosome and specifically C-terminal domains of EngA^MS^ are required to facilitate this interaction. Moreover, EngA^MS^ devoid of N-terminal region interacts well with 50S even in the absence of GTP, indicating a regulatory role of the N-terminal domain in EngA^MS^-50S interaction.

## Introduction

The P-loop NTPases constitute a family of proteins characterized by a common core structure which responds to nucleoside triphosphate (NTP) binding and hydrolysis by acquiring altered conformations. The P-loop NTPase fold is the most common protein fold in all life forms [1] and is characterized at the sequence level by a typical N-terminal Walker A motif GxxxxGK[ST], which is required to correctly orient the triphosphate moiety of the nucleotide [2], [3], [4]. At the distal end of P-loop, Walker B motif contains a conserved aspartate residue that binds a Mg^++^ ion [4].

The GTPase superclass belongs to one of the seven monophyletic lineages within P-loop NTPase fold which comprises of proteins that specifically bind and hydrolyze a small signaling molecule, guanosine triphosphate (GTP) [5]. Structurally, GTPases exhibit distinct motifs namely G1, G2, G3, G4 and G5. Both the G1 and G3 motifs overlap with Walker A and Walker B motifs, respectively, whereas G2 belongs to the loop forming switch I. G4 has a characteristic [NT]KxD sequence which is unique to GTPases and provides specificity to GTP, whereas G5, which comprises of SA[KL] is not characterized yet [6]. Based on the phylogenetic analysis by Leipe et al., P-loop GTPases are divided into two classes: the TRAFAC (designated after translation factor-related) and the SIMBI (after signal recognition GTPases, the MinD and the BioD superfamily) [5]. Each of the two GTPase classes is further subdivided into different superfamilies and families based on the domains' architecture. For example, TRAFAC class is subdivided into five superfamilies viz.: translation factor superfamily, OBG-HflX-like superfamily, TrmE-Era-EngA-YihA-Septin like superfamily, Ras-like superfamily, and Myosin-kinesin superfamily. Similarly, the SIMIBI class is comprised of MinD/Mrp-ETK superfamily and BioD-FTHFS superfamily [5].

Broad conservation and essential characteristics of the P-loop GTPases suggest that they play a major role in microbial physiology [7], [8], [9]. For example, Obg which was initially discovered in *Bacillus subtilis*, regulates sporulation, chromosome partitioning and replication, and mycelium development (as reviewed in [5], [7], [8], [9], [10]). Interestingly, it was reported that ObgE of *E. coli* binds to ppGpp, which is known as a stress response regulator produced in bacterial cells as a consequence of amino acid starvation [11]. These findings thus suggest that Obg plays a role in stress response. Another GTPase, namely Era binds to RNA and regulates chromosomal segregation, cell cycle and metabolism [5], [7], [8], [9], [10]. In a recent study it was observed that Era of *E. coli* interacts with MazG, however no further implication of this interaction was reported [12]. ThdF/TrmE is another Era-like GTPase which plays an important role in protein synthesis. Mutation in the gene encoding ThdF/TrmE results in hypo-modified tRNA and extensive frame-shifting during protein synthesis [13], [14].

EngA family of GTPases belong to TrmE-Era-EngA-YihA-Septin like superfamily of TRAFAC class and EngA and its orthologues are only members of the superfamily that are known to contain two GTPase domains [5], [10], [15]. The *engA* gene is essential in *Nesseria gonorrhoeae* [15], *E. coli* [16] and *B. subtilis* [17] and the *engA* depletion mutants exhibit unusual phenotypes such as filamentation, aberrant chromosomal segregation, alteration in cell shape and abnormal polysome profiles [16], [17]. There are evidences which suggest role of these GTPases in ribosome assembly and stability [18], [19], [20], [21], [22], [23]. It was observed in a genetic screen that two genes encoding universal GTPases, ObgE and EngA restore the loss of phenotype due to deletion of an unrelated gene *rrmJ*, which encodes a heat-inducible RNA methyltrnsferase [22]. RrmJ is involved in methylation of 23S ribosomal RNA (rRNA) at U2552 position and deletion of *rrmJ* causes disruption of ribosome biogenesis [22].

*Mycobacterium tuberculosis* is a slow-growing human pathogen which can persist in the host for years, often leading to latent tuberculosis (TB) upon infection which is difficult to treat. In addition, there are certain types of bacterial population known as persisters that survive despite the use of antibiotics [24]. To eradicate the latent and the persistent bacilli, a continuous multi-drug therapy for 6–9 months is recommended. However, non-compliance with multidrug prolonged therapy is a major cause of the development of multi- and extensively drug resistant (commonly known as MDR and XDR, respectively) strains of *M. tuberculosis*. The MDR and XDR strains are resistant to majority of the first and second-line anti-TB drugs, and are extremely difficult to treat with the current agents [24], [25]. According to World Health Organization, ∼1.5 million people succumbed to tuberculosis in a single year in 2010 [25]. *M. tuberculosis* has globally infected over 9 million individuals, of that ∼5% are cases of the MDR-TB [25]. These statistics thus clearly demonstrate that an effective short term chemotherapeutic option is urgently required to treat drug-resistant TB cases. The EngA GTPase being essential in most of the microorganisms and being absent in humans offers an attractive target to explore for the development of new antimicrobials. The genome sequence analysis of *M. tuberculosis* demonstrates the presence of an open reading frame (ORF) encoding *engA* homologue, which is highly conserved across other species. However, an exact role of EngA in mycobacteria is not known, which prompted us to perform the present study.

Here we characterize the function of EngA protein in mycobacteria by using an EngA homologue from *Mycobacterium smegmatis* (termed as EngA^MS^) encoded by an ORF namely *MSMEG_3738*. Our study establishes that EngA^MS^ is a functional GTPase which is associated with ribosomes and regulates the assembly of ribosomal subunits in the cell. By performing deletion and mutagenesis studies with EngA^MS^, we dissect the role of two GTPase domains in the interaction of EngA^MS^ with ribosome.

## Results

### Phylogenetic analysis of EngA proteins

EngA constitutes a unique family of GTPases that contains two sets of GTPase motifs, as identified by Bourne *et al.* [6]. EngA was first identified in *Neisseria gonorrhoeae* and named after essential neisserial GTP-binding protein A [15]. Though EngA was identified in mycobacteria by a comparative genome analysis using *E. coli* counterpart as a template [10], and has recently been characterized biochemically [26], a thorough analysis of its function in mycobacteria is yet to be performed.

In order to characterize the role of EngA protein in mycobacteria, a blastp search analysis was conducted by employing a sequence of putative EngA homologue from *M. smegmatis*, MSMEG_3738, against the known microbial sequences. As mentioned in supplementary table 1, MSMEG_3738 homologues were identified primarily from Gram positive organisms, seventeen of which correspond to different mycobacterial species (Table S1). Interestingly, the blastp search revealed that MSMEG_3738 exhibits similarity with several bi-functional GTPases of corynebacteria comprising of cytidylate kinase and GTP-binding activities (Table S1). A thorough analysis of *MSMEG_3738* locus identifies a neighboring ORF *MSMEG_3739* encoding putative cytidylate kinase (Fig. S1), which suggests that not only the *MSMEG_3738* but also some other ORFs in its locus are conserved across different microorganisms. Interestingly, different mycobacterial species exhibit a highly conserved gene organization in *MSMEG_3738* locus, as shown in figure S1. *MSMEG_3738* and its orthologues in other mycobacterial species are preceded by ORFs encoding cytidylate kinase (*cmk*), ribosomal large subunit pseudouridine synthase B (*rluB*), segregation and condensation protein B (*scpB*), and segregation and condensation protein A (*scpA*), respectively.

Next, an unrooted phylogenetic tree was constructed from an alignment of MSMEG_3738 with the orthologous sequences by using neighbor joining method [27], as described in the materials and methods section. It was observed that MSMEG_3738 is clustered in the phylogenetic tree with putative EngA proteins of other mycobacterial species. Interestingly, MSMEG_3738 exhibits closer homology with the proteins of fast-growing mycobacteria such as *M. gilvum* and *M. vanbaalenii* compared to those of slow growing species including *M. leprae*, *M. tuberculosis*, *M. avium* etc. (Fig. S2). Moreover, the corresponding proteins from closely related streptomyces species are distantly located while those from corynebacteria are placed in between these two clusters (Fig. S2).

The amino acid sequence comparison of MSMEG_3738 with other mycobacterial proteins suggests that despite showing divergence in phylogenetic tree, the putative EngA orthologues from fast and slow growing mycobacteria share ∼60% identical residues that constitute two GTPase domains (G-domains) typical to EngA class of GTPases [10] (Fig. S3). Each G-domain contains conserved residues organized in five distinct motifs numbered G1–G5, as described by Bourne *et al.* [6]. Sequence alignment of putative mycobacterial EngA homologues demonstrates conserved sequences in G-domain 1 that include GRPNVGKS, DIPGVT, DTGG, NKVD and SAM corresponding to G1 (GxxGxGKS), G2 (D-(X)_n_-T), G3 (DxAG), G4 (NKxD) and G5 (SA[KL]) motifs, respectively. Similarly conserved motifs were also identified in G-domain 2 of these GTPases corresponding to G1 (GKPNVGKS), G2 (DVAGTT), G3 (DTAG), G4 (NKWD) and G5 (SAK), respectively (Fig. S3). Additionally, both the G-domains also contain intervening loops corresponding to switch regions I and II that are placed adjacent to G2 and G3 domains respectively; these loops provide characteristic fold to the GTPase domains [28]. Further analysis of the amino acid sequences of these proteins indicates that the average molecular mass of the putative mycobacterial GTPases is 50.76±0.56 kDa and the frequency of hydrophobic amino acids is significantly higher (41±1.2%) in comparison to other types of amino acids. The isoelectric points of these proteins are remarkably variable which ranges between 5.6 and 9.0 (Table 1).

**Table 1:** Analysis of putative EngA proteins of mycobacteria.

Source	Molecular Mass	% Amino Acids			IEP
		Charged	Hydrophobic	Others	
Mgi	52	28	40	32	5.6
Mab	51	29	39	32	5.7
Mva	51	28	40	32	6.1
Mycobacterium sp. JDM601	51	29	40	31	6.3
Mpa	50	28	42	30	6.4
Mycobacterium sp. MCS	51	28	40	32	6.4
MSMEG_3738	51	28	40	32	6.5
Mav 104	51	28	43	29	6.7
Map K-10	51	29	43	28	6.8
Map S-397	51	29	43	28	6.8
Min	51	28	42	30	7.2
Mka	50	28	42	30	7.2
Mtu EAS054	50	28	41	31	7.2
Mtu H37Rv	50	28	41	31	7.2
Mle	50	29	41	30	8.5
Mma	51	29	41	30	8.7
Mul	51	28	41	31	9

The biochemical characteristics of MSMEG_3738 and its homologues in different mycobacterial species that include *M. abscessus* (Mab), *M. avium* subsp. *paratuberculosis* K-10 (Map K-10), *M. avium* subsp. *paratuberculosis* S397 (Map S-397), *M. gilvum* (Mgi), *M. intracellulare* (Min), *M. kansasii* (Mka), *M. leprae* (Mle), *M. marinum* (Mma), *M. parascrofulaceum* (Mpa), *M. tuberculosis* EAS054 (Mtu EAS054), *M. tuberculosis* H37Rv (Mtu H37Rv), *M. ulcerans* (Mul), *M. vanbaalenii* (Mva), Mycobacterium Sp. MCS and Mycobacterium Sp. JDM601 were analyzed by using the Vector NTI software. Statistical analysis was performed using Microsoft Excel.

### Molecular modeling of MSMEG_3738

**Figure 1:**
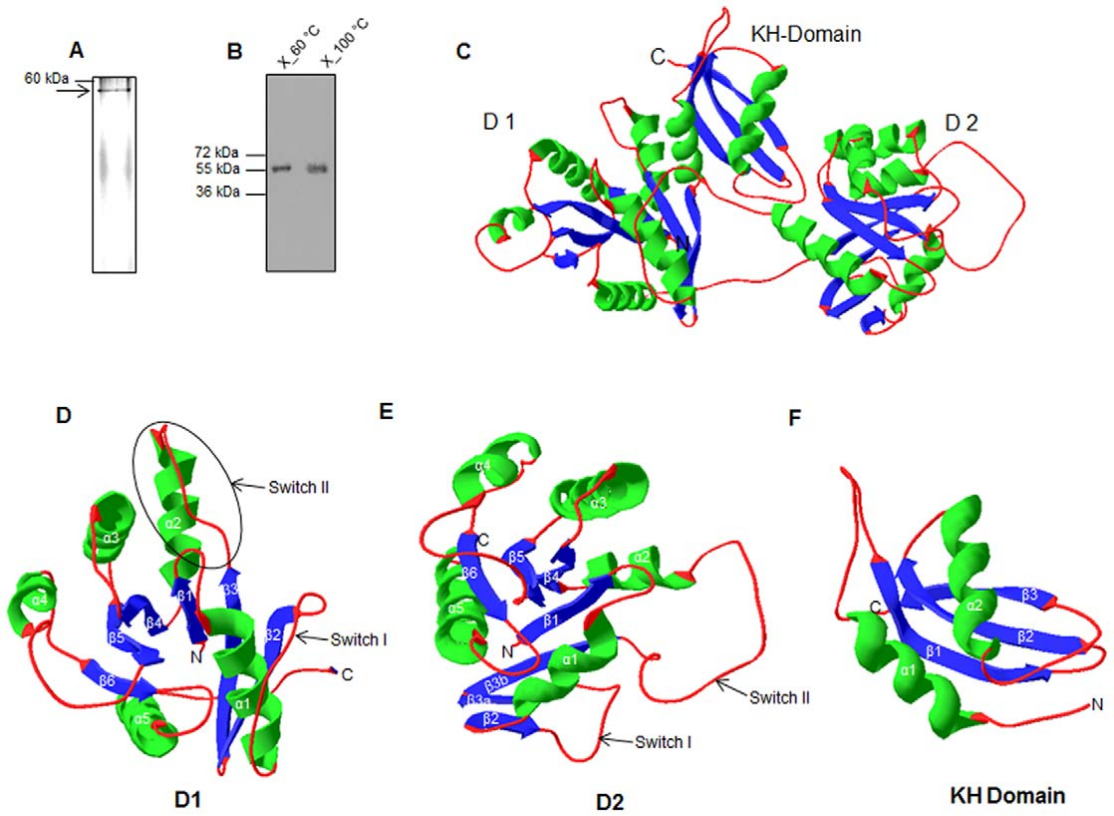
Analysis of MSMEG_3738 modeled structure. A) Fifteen microgram of purified 6×His fusion MSMEG_3738 protein was electrophoresed on 8% native polyacrylamide gel, which was subsequently stained with Coomassie Brilliant Blue R250. The gel shows a single conformation of MSMEG_3738 as marked by arrow. B) *E. coli* BL21 cells transformed with pET28-*EngA^MS^* and induced by IPTG, were cross-linked with 1% formaldehyde as described in materials and methods. Whole cell extracts of cross-linked cells prepared by boiling at 60°C or at 100°C were resolved on 10% denaturing polyacrylamide gel and then transferred to a nitrocellulose membrane which was subjected to immunoblotting by using anti-6×His antibodies. The blot shows that both cross-linked (lane X_60°C) and unlinked (lane X_100°C) proteins migrate at close to the predicted molecular mass of 52.4 kDa. C) Ribbon diagram of MSMEG_3738 three-dimensional model, constructed using SWISS-MODEL Workspace, was prepared by Swiss-PDB Viewer version 4.03. D–F) Elements of the secondary structure that belong to G-domain 1 (D1), G-domain 2 (D2) and KH domain are individually shown for the clarity. Placements of the two switch regions in each G-domain are marked by thin arrows. α-helices, β-sheets and coils are colored in green, blue and red, respectively.

A Protein Data Bank search indicates two EngA homologues, namely, Der in *Thermotoga maritima* (PDB ID: 1MKY) and YphC in *Bacillus subtilis* (PDB ID: 2HJG), for which 3D structures at high resolution are available. In order to understand the tertiary structure of MSMEG_3738, first we sought to determine the oligomeric states of MSMEG_3738. The MSMEG_3738 was overexpressed in *E. coli*, purified with >90% purity as fusion protein containing hexa histidine (6×His) residues at the N-terminus and subjected to native polyacrylamide gel electrophoresis. The gel electrophoresis pattern indicated that the purified MSMEG_3738 preparation contains single conformation, migrating at a position close to 60 kDa (Fig. 1A). Similarly, *in vivo* cross-linking of MSMEG_3738 demonstrates that it migrates on denaturing SDS polyacrylamide gel close to its predicted molecular mass of 52.4 kDa (Fig. 1B). Taken together, these observations suggest that MSMEG_3738 is a monomer. Next, a model of MSMEG_3738 was constructed by using structure of Der protein of *T. maritima* (TmDer) as a template. The modeled MSMEG_3738 exhibits a similar structure to TmDer (Fig. 1C), despite only 33% identity between MSMEG_3738 and TmDer sequences (Fig. S4). Figure 1C shows that the modeled MSMEG_3738 comprises of a typical α/β fold constituting three domains: G-domain1 (D1), G-domain 2 (D2) and a KH domain (Fig. 1C–F). Each G-domain is composed of 5 alpha helices and 6 beta sheets linked by characteristic loops constituting switch I and II. D2 is followed by a third domain namely KH domain consisting of 2 alpha helices and three beta sheets (Fig. 1D–F). Modeled MSMEG_3738 exhibits a cloverleaf structure with the two G-domains D1 and D2 folded over the KH domain (Fig. 1C).

### Biochemical characterization of MSMEG_3738

**Figure 2:**
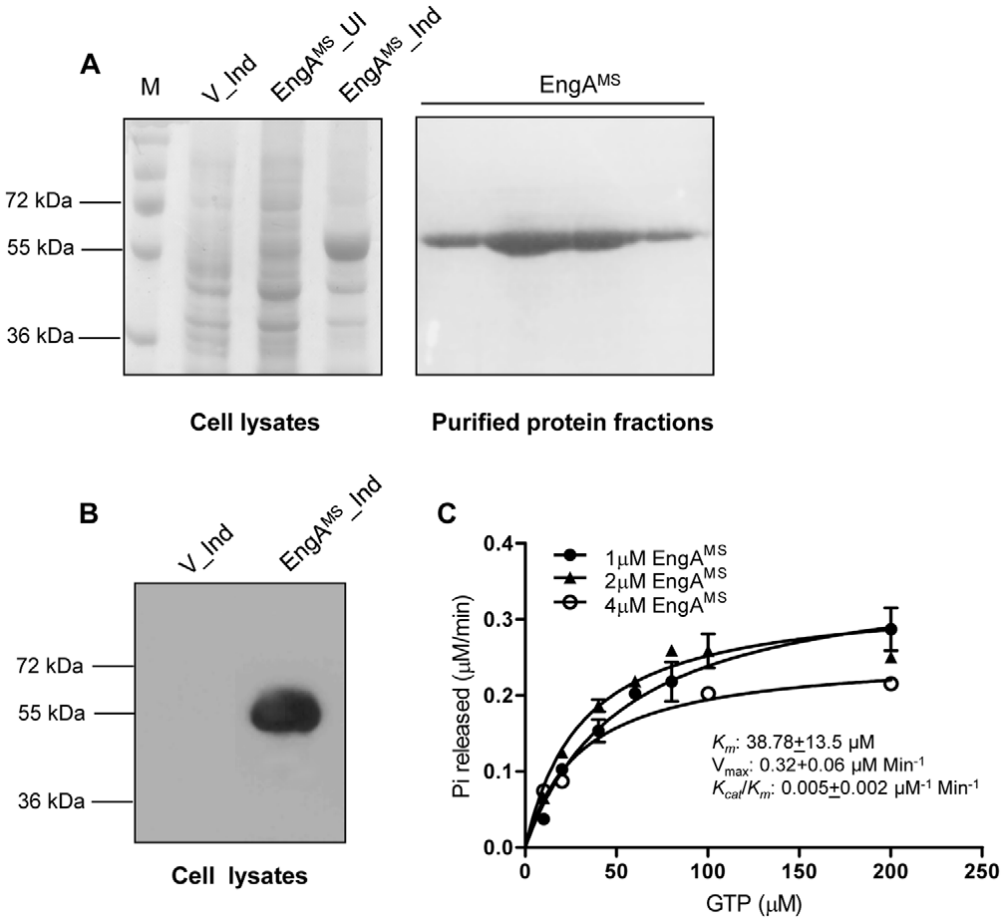
MSMEG_3738 exhibits GTP hydrolyzing activity. A) MSMEG_3738 was expressed in *E. coli* BL21 and purified as 6×His fusion protein, which migrates close to its predicted molecular mass of 52.4 kDa on 10% denaturing polyacrylamide gel. Lanes: M, molecular weight markers; V_Ind, whole cell extracts of IPTG-induced *E. coli* cells transformed with pET28a; EngA^MS^_UI, whole cell extracts of *E. coli* cells transformed with pET*28-EngA^MS^*; EngA^MS^_Ind, whole cell extracts of IPTG-induced *E. coli* cells transformed with pET*28-EngA^MS^*; EngA^MS^, different elution fractions of purified 6×His-EngA^MS^ protein. B) Cell lysates prepared from IPTG-induced *E. coli* BL21 harboring either empty vector pET28a (V_Ind) or pET28- *EngA^MS^* (EngA^MS^_Ind) were resolved on 10% denaturing polyacrylamide gel and then transferred to a nitrocellulose membrane which was subjected to immunoblotting by using anti-6×His mouse monoclonal antibodies. C) One to 4 µM purified MSMEG_3738 was subjected to GTP hydrolysis by incubating with varying concentrations of GTP and the values were plotted in a graph using GraphPad Prism software, as described in the materials and methods section. The x- and y-axes represent GTP concentrations (µM) and rate of Pi release (µM/Min) due to GTP hydrolysis, respectively. Each assay was performed in triplicate and the mean values ± s.d. were used to determine the GTPase activity.

Based on above observations, it is speculated that MSMEG_3738 has a GTP hydrolyzing activity. To confirm this hypothesis, the MSMEG_3738 was overexpressed in *E. coli* and purified as described. Coomassie staining of the denaturing polyacrylamide gel and Immunoblot analysis using anti-6×His antibodies indicate that the MSMEG_3738 was overexpressed to a level of ∼50% of the total soluble proteins specifically in recombinant *E. coli* strains harboring pET28- *EngA^MS^* but not in the cells containing empty vector pET28a after IPTG induction (Figs. 2A–B). The protein was purified with greater than 95% purity which migrates on denaturing SDS polyacrylamide gel close to its predicted molecular mass of 52.4 kDa (Fig. 2A, lanes 5–8). Subsequently, different concentrations of the purified protein were employed in a GTPase assay, which demonstrated that the maximum rate of GTP hydrolysis by MSMEG_3738 is 0.32±0.06 µM Min^−1^. With the observed *K_m_* ^GTP^ of 39 µM, the purified MSMEG_3738 exhibits a turnover of 0.005 Min^−1^ (Fig. 2B). On the contrary, the control experiments conducted either in the absence of MSMEG_3738 or with heat-inactivated enzyme preparation did not result into sufficient GTP hydrolysis (Fig. S5) which demonstrates that in the above reactions GTP was hydrolyzed specifically by the active MSMEG_3738 preparations. Importantly, MSMEG_3738 did not hydrolyze other nucleotides such as CTP or UTP. Based on the above analyses, MSMEG_3738 was referred hereafter as EngA^MS^.

**Figure 3:**
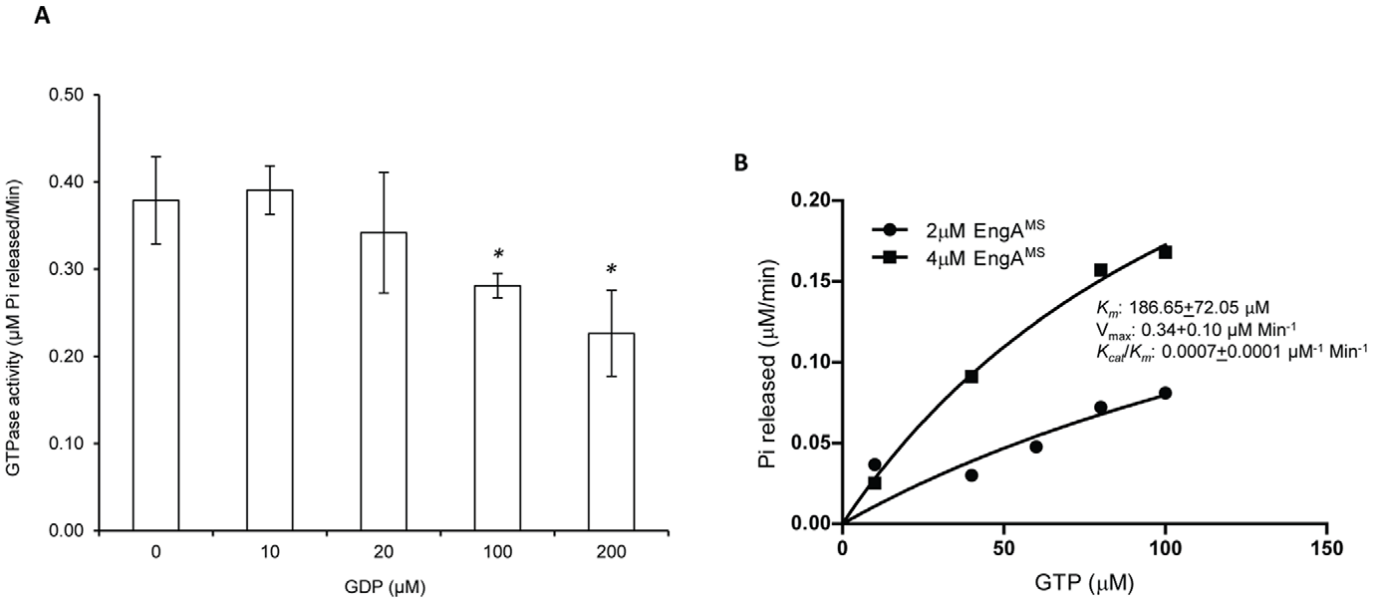
The GTPase activity of EngA^MS^ is inhibited by GDP. A) GTPase assay was performed, as described in the text, by using 1 µM EngA^MS^ in the reaction mixtures each containing 200 µM GTP and different concentrations of GDP. Shown is the bar graph plot using values of the rate of GTP hydrolysis (µM Pi released per minute) and concentrations of GDP (µM), represented on y- and x-axis respectively. B) GTPase assay was performed by using 2–4 µM EngA^MS^ in the reaction mixtures each containing 10 µM GDP and different concentrations of GTP and the values were plotted in a graph using GraphPad Prism software, as described in the materials and methods section. The x- and y-axes represent GTP concentrations (µM) and rate of Pi release (µM Pi released per minute) due to GTP hydrolysis, respectively. Each assay was performed in triplicate and the mean values ± s.d. were used to determine the GTPase activity. *P<0.05.

The GTP hydrolysis by GTPases involves a repeated series of events, each encompassing three different conformations of the protein. Binding of GTP with the protein leads to an ‘active’ conformation, which is subsequently transformed to an ‘inactive’ GDP bound state as a result of GTP hydrolysis. Finally, release of GDP confers an ‘empty’ state which is ready to initiate another cycle. For an efficient GTPase cycle, it is essential that GTP must replace GDP, rather than vice versa. However, several GTPases such as EF-Tu (Elongation Factor Tu) bind GDP rather firmly after hydrolyzing GTP, and for moving the overall reaction in the forward direction, an accessory factor is required which promotes replacement of GDP by GTP [6]. These observations thus suggest that GDP acts as a competitive inhibitor of GTPase activity and the rate of GDP dissociation from the protein is a determinant of its activity [6]. Hence, it was verified whether EngA^MS^ also respond to GDP. A GTPase reaction was setup typically as defined in figure 2, but in the presence of varying concentrations of GDP. Figure 3A demonstrates that the GTPase activity of EngA^MS^ is gradually reduced with the addition of an increasing amount of GDP (Fig. 3A). In the reaction containing 200 µM GTP, the GTPase activity was reduced by 25% and 40% after the addition of 100 µM and 200 µM GDP, respectively (Fig. 3A); though at the same concentrations no effect of ADP was detected on the GTPase activity of EngA^MS^. Measurement of kinetic parameters reveals that in the presence of GDP the *K_m_* ^GTP^ of EngA^MS^ increases to ∼185 µM with a seven-fold reduction in turnover, whereas the maximum rate of GTP hydrolysis is not affected in comparison to ‘no-GDP’ control (Fig. 3B). These results thus clearly indicate that GDP acts as a competitive inhibitor of GTPase activity of EngA^MS^.

### Assessment of EngA^MS^ function in mycobacteria

**Figure 4:**
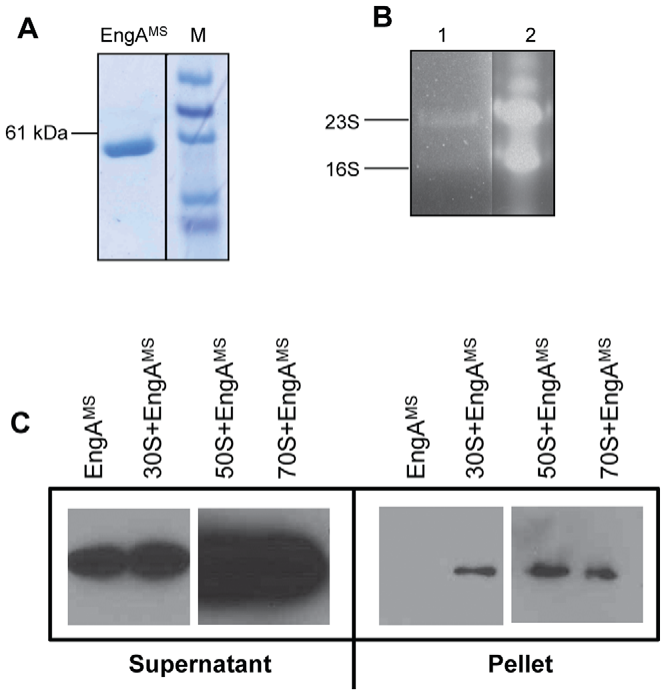
EngA^MS^ co-elutes with ribosomal RNA and interacts with ribosomal subunit proteins *in vitro.* A) The *E. coli* BL21 (DE3) cells overexpressing EngA^MS^ were lysed in RNase-free environment by repeated freeze-thaw cycles and EngA^MS^ was purified as 6×His fusion protein, which migrates close to its predicted molecular mass of 52.4 kDa on 10% denaturing polyacrylamide gel. M, molecular weight markers. B) 16S rRNA and 23S rRNA co-elute with purified EngA^MS^ as shown by resolving the phenol–chloroform extract of purified EngA^MS^ by formaldehyde-agarose gel electrophoresis (Lane 1). The identity of these bands was also verified by reverse transcription-PCR amplification of 23S and 16S-specific sequences using purified protein extract as a template. Ribosomal RNA specifically purified from *E. coli* cells by using Trizol (Invitrogen) was resolved in the adjacent well as a positive control (Lane 2). C) EngA^MS^ interacts with ribosomal subunits purified from *M. smegmatis*. The ribosomal subunits were purified from *M. smegmatis* and subjected to interaction with EngA^MS^ using low salt buffer (30 mM NH_4_Cl) and in the presence of non-hydrolysable GTP analogue GMP-PNP, as described in the materials and methods section. Immunoblots using anti-6×His antibodies, for the supernatant and pellet fractions are shown. Sample containing EngA^MS^ alone was used as a negative control which retained in the supernatant fraction, whereas in the presence of 30S, 50S and 70S specific EngA^MS^ signals were detected from the pellet fraction, thus confirming a specific interaction of EngA^MS^ with all three ribosomal subunits.

EngA is a regulator of multiple biological functions such as cell division, chromosomal segregation and assembly of 50S ribosome [7], [16], [17], [18], [20], [21], [22]. In order to assess whether purified EngA^MS^ is associated with ribosome, the affinity purified recombinant EngA^MS^ was subjected to rRNA extraction which revealed that purified EngA^MS^ preparations indeed contain both the 23S and 16S rRNAs (Fig. 4B, lane 1). Further, specific interaction between purified EngA^MS^ and ribosomal subunit was analyzed *in vitro*. Recombinant 6×His-EngA^MS^ purified from *E. coli* was separately incubated in the presence of non-hydrolysable GTP analogue GMP-PNP, with each of the 30S, 50S and 70S ribosomal subunits isolated from *M. smegmatis* and the EngA^MS^ –ribosome complexes were fractionated by ultracentrifugation as described. Association of EngA^MS^ with a ribosomal subunit was analyzed by immunoblotting using anti-6×His antibody of the pellet fraction containing ribosome-EngA^MS^ complex, whereas free EngA^MS^ was detected in the supernatant fraction. As the strength of ribosomal interactions is inversely correlated to the amount of salt [19], [23], these experiments were performed by using low salt buffer. As shown in figure 4C, a specific signal corresponding to EngA^MS^ is detected in the pellet fraction of each of the samples containing 30S, 50S and 70S ribosomal subunits respectively (Fig. 4C, pellet fractions, lanes 2–4), whereas fractionation of a sample lacking ribosome does not exhibit presence of EngA^MS^ in the pellet fraction (Fig. 4C, pellet fraction, lane 1). In contrast, a specific signal is obtained from the supernatant fraction of all of these samples (Fig. 4C, supernatant fractions, lanes 1–4). These results thus clearly demonstrate that EngA^MS^ interacts with all three ribosomal subunits of *M. smegmatis*.

**Figure 5:**
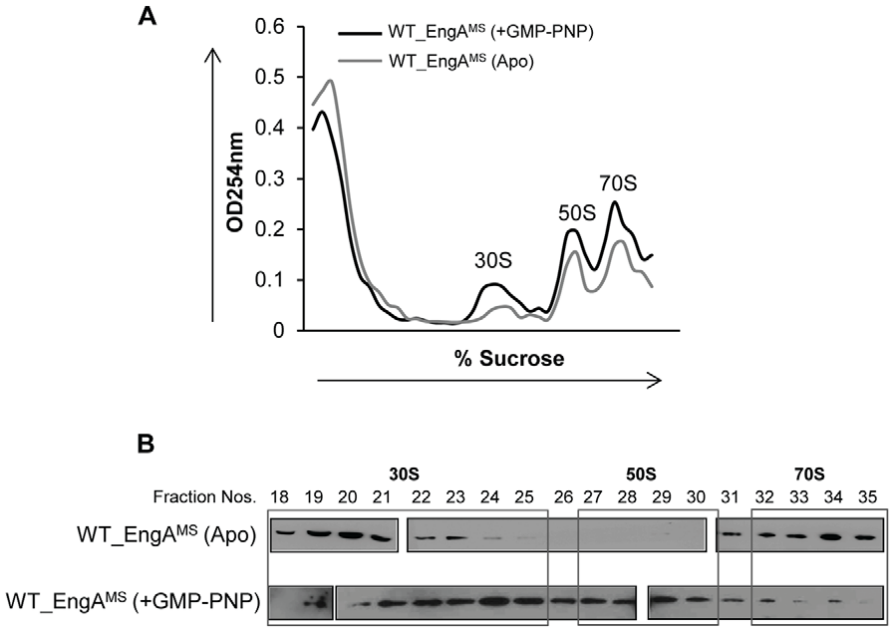
GTP is required for interaction of EngA^MS^ with 50S subunit of ribosome. A) The *E. coli* BL21 (DE3) cells overexpressing EngA^MS^ were lysed in RNase-free environment by repeated freeze-thaw cycles and fractionated by ultra-centrifugation (using Beckman SW28 rotor) in the presence (+GMP-PNP) or absence (Apo) of 1 mM GMP-PNP on 10–45% sucrose gradient prepared in high salt buffer (containing 100 mM NH_4_Cl). Equal fractions of 1 ml each were collected from top to bottom and A_254_ values for all the fractions were plotted in a graph which shows a characteristic profile of different ribosomal subunits. Notably the addition of GMP-PNP results in better assembly of ribosomal subunits as evidenced by more area under the curves corresponding to 30S, 50S and 70S ribosomal subunits, respectively. B) Immunoblotting of the fractions 18–35 containing 30S, 50S and 70S ribosomal subunits, using anti-6×His antibody. EngA^MS^ does not interact with 50S subunit in the absence of nucleotide (Apo, top blot- fractions 27–30). However, the interaction is restored by the addition of GMP-PNP (+GMP-PNP, bottom panel- fractions 27–30).

Next, to verify interactions of EngA^MS^ with ribosomal subunits *in vivo*, EngA^MS^-ribosome co-fractionation experiments were carried out by subjecting the lysate of *E. coli* cells overexpressing 6×His-EngA^MS^ to sucrose density gradient centrifugation. As seen in figure S6, immunoblotting of different ribosome-containing fractions using anti-6×His antibody demonstrates specific signals corresponding to co-fractionated EngA^MS^ (Fig. S6B). Conversely, a control fractionation experiment performed with the purified EngA^MS^ under the similar conditions did not exhibit any signal in the immunoblots of the corresponding fractions (data not shown). These results thus suggest that under *in vivo* conditions EngA^MS^ interacts with ribosomes even in the absence of GTP. However, the subsequent studies indicated that these interactions are not stable since only 30S and 70S subunits remain associated with EngA^MS^ at higher salt concentration (Fig. 5B, top panel), whereas EngA^MS^-50S interaction persists under these conditions only in the presence of GMP-PNP (Fig. 5B, bottom panel). These results thus propose that EngA^MS^ performs differently based on the level of GTP in the cell. It is noteworthy to mention that co-fractionation of lysates in the presence of GMP-PNP results in better yields of 30S, 50S and 70S ribosomes compared to those obtained in the absence of nucleotide (Fig. 5A), which suggests that EngA^MS^ –GTP interaction affects ribosome assembly.

From now on, the co-fractionation experiments were carried out using lysate of *E. coli* cells overexpressing wild-type or mutant derivatives of 6×His-EngA^MS^ in a buffer containing 100 mM NH_4_Cl, and in the presence of 1 mM GMP-PNP whenever indicated.

### Role of G-domains in EngA^MS^-ribosome interactions

**Figure 6:**
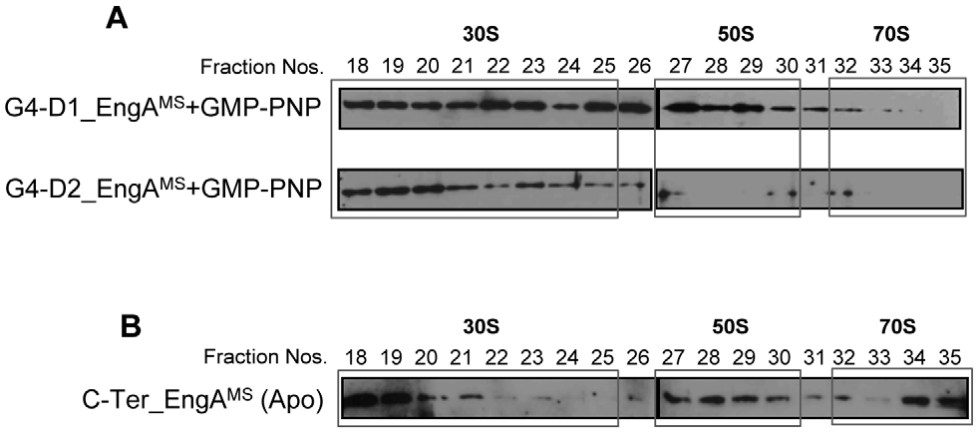
C-terminal domains of EngA^MS^ interact with 50S subunit of ribosome. A) Point mutant derivatives of EngA^MS^ containing D154N (G4_D1) and D331N (G4_D2) substitutions in nucleotide binding motif of both the G-domains were subjected to ribosome co-fractionation experiments in the presence of GMP-PNP using sucrose gradient prepared in high salt, as in figure 5. Immunoblots of the fractions containing 30S, 50S and 70S subunits using anti-6×His antibody indicates that the G4_D2 but not the G4_D1 is required for interaction with 50S ribosomal subunit (fractions 27–30). B) Deletion of 178 amino acid residues from the N-terminal region corresponding to G-domain 1 of EngA^MS^ (designated as C-Ter EngA^MS^) restores the interaction of EngA^MS^ with 50S even in the absence of nucleotide. Shown are the Immunoblots of fractions containing 30S, 50S and 70S subunits prepared by fractionation of *E. coli* lysates containing C-Ter EngA^MS^ as described in materials and methods.

Our observation that at higher salt concentration EngA^MS^ interacts with 50S only in the presence of GTP suggests that binding of guanosine nucleotides to G-domains is critical to EngA-ribosome interactions. In order to understand the mechanism of interaction of EngA^MS^ with ribosome, two individual mutants of EngA^MS^ were constructed- one harboring mutation in the critical GTP-binding motif of D1 (D154N in G4_D1) and the other in D2 (D331N in G4_D2) respectively, and subsequently employed in ribosome interaction studies. These substitutions are known to alter the overall specificity of EngA from GTP to XTP [23], [28], [29]. However, to ascertain the nucleotide-free state, these mutants were subjected to fluorescent nucleotide binding assay using mant-GDP. Fluorescence of the mant-nucleotides is increased after binding with the protein, which is in direct proportion to the amount of binding [30]. As anticipated, both D154N and D331N substitutions result in ∼50% reduction in the binding affinity of EngA^MS^ to the guanosine nucleotide in comparison to the wild-type protein (Fig. S7). Next, fractionation of lysates containing these EngA^MS^ mutants in the presence of GMP-PNP, followed by immunoblotting of the resulting 30S, 50S and 70S ribosomal fractions indicated that 50S ribosomal fractions prepared from G4_D2 mutant is devoid of EngA^MS^-specific signals (Fig. 6A, bottom panel). In contrast, a similar mutation in D1 does not cause any effect on EngA^MS^-ribosome interaction (Fig. 6A, top panel). These results thus point out that G4 motif of D2 but not of D1 is critical to EngA^MS^-50S interaction (Fig. 6A). Taken together, the above observations propose that EngA^MS^ -50S interaction is regulated by the binding of GTP to D2.

### Effects of deletion of N-terminus on EngA^MS^-ribosome interactions

Requirement of a functional D2 domain strongly suggests that the C-terminal region of EngA^MS^ (designated as C-ter EngA^MS^) is important for its interaction with ribosome. In order to determine role of C-terminal domains in EngA^MS^-ribosome interactions, the C-ter EngA^MS^ was individually expressed and strength of its interaction with ribosomal subunits was measured as described above. A 534 bp long sequence from the 5′ end of *MSMEG_3738* encompassing the entire D1 region was deleted and the remaining 882 bp long DNA fragment cloned in pET28a was used to transform *E. coli* BL21 cells, leading to the expression of ∼33 kDa long C-ter EngA^MS^ protein which contain both the D2 domain and the KH domain. The co-fractionation experiments conducted with the lysates containing C-ter EngA^MS^ and subsequent immunoblotting indicates that under *in vivo* conditions, the C-ter EngA^MS^ lacking N-terminal residues associates well with all three ribosomal subunits at higher salt concentration, even in the absence of GTP (Fig. 6B). This is in contrast to the wild-type EngA^MS^ which strictly requires GTP for a stable interaction with 50S ribosome (Fig. 5B, bottom panel). These results thus clearly demonstrate that in the absence of GTP, the N-terminal region of EngA^MS^ imposes a distinct conformation which does not allow EngA^MS^-50S interaction.

## Discussion

In the present study we have characterized the underlying mechanism of EngA function in mycobacteria. It has been reported that the bacterial cells with reduced expression of EngA are compromised for growth, suggesting EngA is essential in bacteria [15], [16], [17], [18]. Genome sequence analysis of *M. smegmatis* suggested that an ORF namely *MSMEG_3738* encodes EngA GTPase (EngA^MS^), which is conserved in other mycobacteria, including *M. leprae*, a mycobacterial species which has lost 33% of its genome compared to other mycobacteria of *M. tuberculosis* complex (Fig. S2). This indicates that the EngA protein might play an important role in the physiology of mycobacteria. Importantly, conservation of *engA* locus in mycobacteria (Fig. S1) suggests that the respective proteins may act synergistically and warrants further studies to characterize the significance of this genetic arrangement in genus Mycobacterium.

In order to understand the tertiary structure of EngA^MS^, we constructed a model of EngA^MS^. Electrophoresis pattern of the purified EngA^MS^ on native polyacrylamide gel and *in vivo* cross-linking [31] of EngA^MS^ in *E. coli* overexpression strain confirmed that EngA^MS^ is indeed a monomer (Fig. 1A–B). For any two proteins, a sequence identity greater than 25% predicts similar three-dimensional structures [32]. Hence, we used a template Der protein of *T. maritima* (TmDer), with which EngA^MS^ shares ∼33% identity, to construct the model of EngA^MS^. The model of EngA^MS^ was validated by constructing and analyzing the Ramachandran plot which indicated >90% residues are in the allowed region. Molecular modeling of EngA^MS^ predicts α/β folds (Fig. 1C–F) similar to TmDer [28], which strongly advocates a GTPase activity in EngA^MS^. In agreement to this hypothesis, we observed a substantial GTPase activity in EngA^MS^ (Fig. 2), which was ∼4-fold higher in comparison to TmDer, as reported by Robinson *et al.* [28]. These results thus demonstrate that functionally the mycobacterial proteins differ from TmDer. A multiple level of regulation is proposed for EngA GTPases due to several inter-domain interactions [28]. A thorough analysis of the amino acid sequence alignment of EngA proteins of *M. smegmatis* and *T. maritima* designates certain differences at critical positions that are known to determine the folding of D1 and D2 over KH domain [28]. For example, EngA^MS^ lacks residues corresponding to C57, S367, Y328 and Y356 in TmDer which establish interactions of D1 and D2 with KH domain [28]. In addition, EngA^MS^ contains a stretch of amino acid residues at the extreme N-terminus preceding the G1_D1 motif that is specifically present in mycobacteria but is absent in TmDer (Figs. S3, S4). Taken together these observations indicate that though EngA^MS^ exhibits similar arrangement of molecules to TmDer, both the proteins have acquired different types of inter-domain interactions causing divergence in their activities. A careful analysis of EngA^MS^ model indeed corroborates these observations, which designates involvement of residues different from those reported in TmDer for causing potential interactions between GD1-KH and GD2-KH, respectively (Fig.S8). Based on proximity of residues in modeled EngA^MS^, we envisage interaction of D_89_-T-G-G_92_ of G3_D1 with G_389_-R-L-N-T-F-L-K-E_397_ of KH domain, and N_328_-K-W-D_331_ of G4_D2 with S_361_-A-L-T-G-R-A_367_ of KH domain (Fig. S8 (insets)). Importantly, several of these residues are critical for EngA^MS^ GTPase activity. The G3_D1 is adjacent to switch II which provides characteristic folding to create active sites, whereas G4_D1 provides specificity to GTP [6]. Thus it is tempting to speculate that GTP binding causes a conformational change in EngA^MS^ which subsequently affects the protein's function. In an ongoing study we are characterizing the role of these residues in the proposed inter-domain interactions in EngA^MS^ and their subsequent effect on its GTPase activity.

In order to understand the role of EngA in mycobacteria we sought to determine whether EngA^MS^ interacts with ribosome and affects ribosome assembly, as reported [18], [20]. A GTPase Era of EngA superfamily, which contains conserved RNA-binding motifs in the C-terminal KH domain, binds with RNA [33]. Though, EngA^MS^ co-purifies with rRNAs (Fig. 4A), the absence of distinctive RNA recognition elements in the KH domain of EngA^MS^ rules out its direct association with the rRNAs. Conversely, our observations that EngA^MS^ is co-sedimented with purified ribosomes of *M. smegmatis* during ultracentrifugation (Fig. 4B) suggest that EngA^MS^ directly interacts with ribosomal subunit proteins and traces of rRNAs packed with the ribosome are eluted during purification of EngA^MS^. Interaction of EngA^MS^ with ribosome was also verified *in vivo* using *E. coli* overexpressing 6×His-EngA^MS^ (Fig. S6). An ability of EngA^MS^ to recognize *E. coli* ribosome also suggests that not only the tertiary structure of these proteins but also the mode of their interaction is highly conserved. Sensitivity of EngA^MS^-50S complex to salt however, indicates that EngA^MS^ establishes a weak interaction with 50S and additionally requires GTP to maintain a stable interaction. Conversely, interaction of EngA^MS^ with other two ribosomal subunits 30S and 70S is not affected by the presence of nucleotides. This is in contrast to EngA of *E. coli* which distinctly requires binding of GDP to D1 and GTP to D2 for interaction with all three ribosomal subunits [23]. The two G-domains in EngA exhibit antagonistic GTP binding and hydrolysis activities [28], which indicates distinct requirement of each of these domains in determining the EngA activity. Strikingly, D154N mutation in G4_D1 did not affect EngA^MS^-50S interaction (Fig. 6A, top panel), though the mutant was defective in nucleotide binding activity (Fig. S7). On the contrary, similar amino acid substitution in G4_D2 at position 331 caused cessation of EngA^MS^-50S interaction (Fig. 6A, bottom panel) suggesting that ribosomal subunits bind with EngA^MS^ primarily at the C-terminal region. This was further substantiated by co-fractionation of truncated EngA^MS^ containing isolated C-terminal region, with 50S at high salt concentration and in the absence of GTP- the conditions that do not favor the wild-type EngA^MS^-50S interaction (Figs. 5B and 6B). These results thus demonstrate that in the absence of GTP, the N-terminal region probably imparts an unfavorable state to EngA^MS^ which prevents its interaction with 50S ribosomal subunit. Notably we also observed that G4_D2 mutant does not fractionate with 70S (Fig. 6A, bottom panel). We reasoned that it is due to poor assembly of 50S in *E. coli* due to overexpression of G4_D2 mutant derivative of EngA^MS^, which subsequently affects the assembly of functional 70S ribosome. Furthermore, our observation that mutations in both the G-domains do not affect interaction of EngA^MS^ with 30S subunit warrants further studies to characterize the role of KH domain in establishing this interaction.

**Figure 7:**
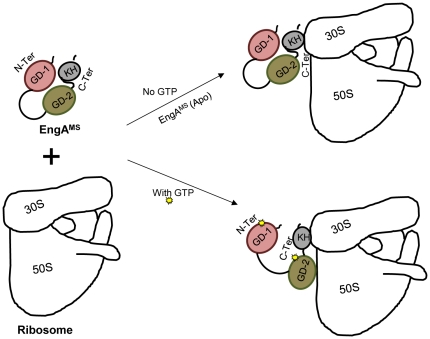
Interaction of EngA^MS^ with ribosomal subunits is regulated by GTP. A hypothetical model is shown depicting the mechanism of regulation of EngA^MS^- ribosome interaction by GTP. Wild-type EngA^MS^ in the apo form interacts only with 30S subunit due to its inaccessibility to 50S. However, upon binding with GTP, the EngA^MS^ changes its conformation by an unknown mechanism so that its C-terminal domains are accessible to interact with 50S.

In conclusion, we have characterized the role of EngA GTPase in mycobacteria by employing *M. smegmatis* protein encoded by *MSMEG_3738*. Our results suggest that *MSMEG_3738* product, which we named as EngA^MS^ is a functional GTPase that regulates ribosomal assembly. Further we demonstrated that the individual G-domains play specific roles in establishing EngA^MS^ interaction with ribosome, particularly with 50S subunit: the C-terminal region provides a platform to organize EngA^MS^-ribosome interaction, whereas the N-terminal region plays a regulatory role in response to a signal provided by GTP (Fig. 7). At present we are in a process of conducting further studies to better understand the molecular mechanism of inter-domain interactions in EngA^MS^ and their subsequent effect on its association with ribosome. Since EngA GTPase is essential in mycobacteria and is absent in humans, these studies will certainly help designing novel targets for tuberculosis drug discovery.

## Materials and Methods

### Bacterial strains, culture conditions and plasmid

Bacterial strains used in this study are listed in supplementary table 2. Luria-Bertani (LB) broth and LB agar were the general-purpose media for culturing *E. coli*, and Middlebrook 7H9 broth supplemented with 1× OADC, 0.05% Tyloxapol and 0.5% glycerol, and Middlebrook 7H10 agar supplemented with 1× OADC and 0.5% glycerol for culturing *M. smegmatis*. Both *E. coli* and *M. smegmatis* were grown at 37°C with shaking at 200 RPM. When required, 50 µg/ml kanamycin was added.

### Cloning, expression and purification of proteins

Wild-type *MSMEG_3738* ORF was PCR amplified from the genomic DNA using primer pairs MS3738_F and MS3738_R (Table S2), designed in a way such that the PCR amplicon carried an *Nde* I site at the 5′ end and a *Hind III* site at the 3′ end. PCR was performed using a mixture of Taq polymerase (Invitrogen) and Pfu polymerase (Stratagene) according to the manufacturer's specifications. Each of the 30 cycles was carried out at 94°C for 15 sec, 55°C for 2 min, and 72°C for 1 min, followed by final extension at 72°C for 10 min. Subsequently the *MSMEG_3738* ORF was cloned in pET28a (Table S2) by implementing the standard molecular biology techniques [34] resulting in the pET*28-EngA^MS^* construct. Point mutation derivatives of *MSMEG_3738* harboring substitutions D154N and D331N were generated by the help of Quick-Change site directed mutagenesis kit (Stratagene), using 100 ng pET*28-EngA^MS^* construct as a template, and primer pairs D1_F-D1_R, and D2_F-D2_R respectively (Table S2), according to the users' manual. Substitution of nucleotides at each position was confirmed by sequencing. For N-terminal deletion, an *Nde* I site was created in pET*28-EngA^MS^* construct after 534^th^ base from the 5′ end of *engA^MS^* ORF by site directed mutagenesis using primers Cter_F and Cter_R (Table S2). The resulting construct was digested with *Nde* I to remove the 534 bp long DNA fragment from the 5′ end encoding N-terminal region of EngA^MS^, and the rest of the DNA was subsequently purified and subjected to self-ligation. The self-ligated construct pET*28- Cter-EngA^MS^*, harboring the truncated *MSMEG_3738* ORF encoding C-terminal region of EngA^MS^ (Cter-EngA^MS^) was subsequently used to achieve expression of 6×His-Cter-EngA^MS^.

For protein expression, both the wild-type and mutant pET*28-EngA^MS^* constructs were used to transform *E. coli* BL21(DE3), according to the standard protocol [34]. *E. coli* transformants were subsequently grown to A_600_ of 0.6–0.8 in LB medium at 37°C followed by incubation at 18°C with 1 mM IPTG for 20 hr. For purification, cells were harvested by centrifugation at 5000*×*g for 5 min at 4°C followed by washing with buffer A (40 mM Tris-Cl pH 8.0, 2 mM MgCl_2_, 20% glycerol and 10 mM Imidazole). Cell suspension in the buffer B (buffer A containing 1 mg/ml lysozyme and 1 mM PMSF) was prepared and subjected to lysis by sonication. EngA^MS^ with histidine tags at the N-terminus was purified by Ni-NTA affinity chromatography according to the manufacturer's protocol (Qiagen). Purity of the protein was analyzed by SDS–PAGE.

### Database search of EngA homologues

EngA sequences were obtained from non-redundant protein sequence database of the National Center for Biotechnology Information (NCBI) by blastp search (http://blast.ncbi.nlm.nih.gov/Blast.cgi) using MSMEG_3738 sequence of *M. smegmatis* (GenBank Accession No. YP_888037.1). Search was performed with sequences of a total of 1775 microbial genome by limiting the number of database sequences for which high-scoring segment pairs (HSPs) will be reported to 100, and the statistical significance threshold for reporting matches against database sequences such that matches expected to be found merely by chance to 10. The description of EngAs used in the analysis and their corresponding GenBank accession numbers are presented in Table 1.

### Phylogenetic analysis

To study the evolutionary relationships among 100 EngA proteins from different organisms, we conducted Phylogenetic analysis using the Vector NTI software (Invitrogen) based on the Neighbor-Joining method [27].

### Multiple sequence alignment

Multiple sequence alignment was performed using the ClustalW algorithm of the AlignX program of the Vector NTI software (Invitrogen) according to the manufacturer's recommendations.

### *In vivo* cross-linking of EngA^MS^

*In vivo* cross-linking was performed typically as described earlier [31]. Briefly, EngA^MS^ was expressed in *E. coli* as described above and the cell pellet from 25 ml culture was washed twice with 1×PBS, and incubated with 1% formaldehyde for 2 hr at 25°C without shaking. The cross-linked samples were harvested by centrifugation and washed once with 1 ml of cold 1×PBS. The cell pellets were resuspended in 40 µl of SDS loading dye, heated at 60°C for 10 min, and centrifuged for 1 min. Aliquots of 10–15 µl of the supernatant were resolved on denaturing polyacrylamide gel followed by immunoblotting by using anti-6×His antibodies, according to the manufacturer's specifications (GE Healthcare Life Sciences). Cross-linking was reversed by incubating the sample in SDS loading dye at 100°C for 20 min.

### Immunoblot analysis

Whole cell extracts of IPTG-induced *E. coli* BL21 strain was prepared from 1 ml culture at A_600_ of ∼2. The cell pellet was washed twice with 1 ml of 1×PBS, lysed in 75 µl of SDS loading dye at 95°C for 10 min and centrifuged for 10 min. Aliquots of 25 µl of the supernatant were resolved on 10% denaturing polyacrylamide gel, and transferred onto Hybond-ECL nitrocellulose membrane (GE Healthcare Life Sciences). To perform immunoblotting shown in figure 2B, the membranes were blocked in 1×PBS with 0.05%Tween 20 (PBST) supplemented with 10% non-fat dried milk and then incubated with primary antibodies against 6×His raised in mouse (GE Healthcare Life Sciences). The blots were thoroughly washed with PBST and incubated with a secondary antibody, HRP-conjugated anti-mouse (GE Healthcare Life Sciences) in PBST. The bound antibodies were detected using SuperSignal West Pico Chemiluminescent Substrate (Thermo Scientific).

### Molecular modeling

Homology model of MSMEG_3738 was constructed using structure of an EngA homologue of *Thermotoga maritima*, Der (TmDer) as a template [28]. TmDer is the first protein of EngA family for which a structure was determined at a high resolution. Though, our database search omitted the TmDer from the analysis because of stringent search parameters, a separate alignment of MSMEG_3738 and TmDer indicates >30% identity (Fig. S3). The model was generated with the help of automatic modeling mode of SWISS-MODEL Workspace (http://swissmodel.expasy.org/workspace) and further analysis was carried out by using Swiss-PDB Viewer version 4.03. Accuracy of the model was evaluated by using Ramachandran plot which was generated by Swiss-PDB Viewer.

### Determination of GTPase activity

GTPase activity was determined by incubating a constant protein concentration with GTP in a reaction buffer (40 mM Tris-Cl pH 8.0, 2 mM MgCl_2_, and 40% glycerol) for one hour. The GTPase activity was expressed as the rate of inorganic phosphate (Pi) release from GTP in the presence of purified protein. The Pi was estimated by a colorimetric method, as described [35]. A separate reaction was setup without any protein to estimate the amount of GTP hydrolyzed during the time of the reaction. This was subtracted from the respective measurement to estimate the amount of Pi released due to GTPase activity. The assay was performed in triplicates and the mean values were used to determine the GTPase activity. To determine kinetic parameters such as V_max_, *K_m_* and *k*_cat_/*K_m_*, the GTPase assay was setup by using increasing substrate concentrations and the rates of Pi release were subsequently plotted against the corresponding substrate concentrations using GraphPad Prism 5.0 software.

### EngA^MS^-RNA co-purification

*E. coli* BL21 (DE3) cells overexpressing EngA^MS^ were suspended in the buffer B and lysed by repeated freeze-thaw cycles. Lysates treated with DNase I were subjected to Ni-NTA affinity chromatography purification, as described above. Fractions containing the purified protein were pooled and stored in aliquots at −70°C. Co-eluted rRNA was recovered from the purified EngA^MS^ by subjecting an aliquot of purified protein to phenol extraction, followed by a phenol-chloroform extraction. The rRNA samples were subsequently analyzed on 0.8% formaldehyde-agarose gel alongside a purified *E. coli* rRNA as a control.

### EngA^MS^-ribosome co-fractionation

EngA^MS^-ribosome co-fractionation experiment was performed with lysates of the *E. coli* cells overexpressing wild-type and mutant derivatives of EngA^MS^. Briefly, lysates were prepared in RNase-free environment by repeated freeze-thaw cycles using ribosome purification buffer (10 mM MgCl_2_, 20 mM Tris-Cl pH 8.0) containing either low salt (30 mM NH_4_Cl) or high salt (100 mM NH_4_Cl) as mentioned in the text, followed by centrifugation at 27000*×*g for 20 min at 4°C. Lysates treated with DNase I were layered on 35 ml sucrose gradient (10–45%) prepared in the ribosome purification buffer in the presence or absence of 1 mM GMP-PNP, subject to reaction conditions. Gradient was subjected to ultra-centrifugation using SW28 rotor (Beckman) at 26000 RPM for 16 hr. Thirty five equal fractions of 1 ml each were collected manually from top to bottom and A_254_ of each fraction was measured. Subsequently, rRNA was extracted from an aliquot of each fraction, and based on rRNA profiles we assigned fraction numbers 18–25 to 30S ribosome, 27–30 to 50S ribosome and 32–35 to 70S ribosome. Aliquots of each of these fractions were subjected to TCA precipitation and were resolved by SDS–PAGE followed by immunoblotting using anti-6×His antibodies (GE Healthcare Life Sciences), as all variants of EngA described here contain an N-terminal 6×His fusion tag.

### *In vitro* EngA^MS^-ribosome binding

For *in vitro* binding of EngA^MS^ with ribosome, ribosomes were purified from *M. smegmatis*. For this, cell pellets from 100 ml culture at A_600_ of 1.0 were suspended in 1 ml low salt ribosome purification buffer (10 mM MgCl_2_, 20 mM Tris-Cl pH 8.0 and 30 mM NH_4_Cl) and subjected to lysis by bead beating for 10 cycles, each for 30 sec with 1 min incubation on ice after every cycle. Lysates treated with DNase I were layered on sucrose gradient and subjected to ultracentrifugation, as described above. The peak fractions corresponding to various ribosomal subunits were pooled, concentrated and stored at −70°C for further use.

For interaction studies, 100 pmol purified protein was incubated with five A_254_ purified ribosomes in the presence of 1 mM GMP-PNP in 100 µl reaction volume containing low salt ribosome purification buffer, for 30 min on ice. To analyze interaction with 50S and 70S subunits, the reaction mixtures were layered on 25% sucrose in low salt ribosome purification buffer, whereas for 30S interaction studies, the reaction mixtures were layered on 15% sucrose-containing buffer. The samples were subjected to ultracentrifugation using S55S rotor (Hitachi) at 40000 RPM for 2 hr. Presence of EngA^MS^ protein in the supernatant and pellet fractions was subsequently analyzed by immunoblotting using anti-6×His antibodies. Purified EngA^MS^ protein alone was used as a negative control.

### Fluorescent nucleotide binding assay

Affinity of guanosine nucleotides towards EngA^MS^ was determined by fluorescent nucleotide binding assay, as described ([23] and reference within). Protein- mant-nucleotide complexes were generated by incubating 4 µM of the wild-type or mutant derivatives of EngA^MS^ proteins with 1 µM mant-GDP (Invitrogen) in a reaction buffer (40 mM Tris-Cl, pH 8.0, 2 mM MgCl_2_, and 40% glycerol) at room temperature for 10 min and 30 min, respectively. Fluorescent intensities of the N-methyl-3′-O-anthranoyl (mant) group attached to the nucleotides were monitored in a 96-well plate by using Synergy HT multi-mode microplate reader (BioTeck) at an excitation wavelength of 360±40 nm and emission wavelength of 440±40 nm. The relative binding affinities of GDP were calculated by comparing the fluorescent intensities following incubation of mant-GDP with WT and mutant proteins.

## Supporting Information

Figure S1 **Phylogenetic tree analysis of microbial EngA proteins.** An unrooted Phylogenetic tree was constructed from an alignment of MSMEG_3738 with the orthologous sequences as listed in Table 1, by using neighbor joining method [27]. The numbers in the parenthesis next to each organism represent the calculated distance values that reflect the degree of divergence between all pairs of sequences analyzed.
(TIF)

Figure S2 **Alignment of MSMEG_3738 with EngA protein sequences of other mycobacterial species.** Homologues of MSMEG_3738 were identified by blastp homology searches in different mycobacterial species that include *M. abscessus* (Mab), *M. avium* subsp. *paratuberculosis* K-10 (Map K-10), *M. avium* subsp. *paratuberculosis* S397 (Map S-397), *M. gilvum* (Mgi), *M. intracellulare* (Min), *M. kansasii* (Mka), *M. leprae* (Mle), *M. marinum* (Mma), *M. parascrofulaceum* (Mpa), *M. tuberculosis* EAS054 (Mtu EAS054), *M. tuberculosis* H37Rv (Mtu H37Rv), *M. ulcerans* (Mul), *M. vanbaalenii* (Mva), Mycobacterium Sp. JDM601and Mycobacterium Sp. MCS, and aligned using AlignX program of Vector NTI software as described in materials and methods section. The number in parentheses before each sequence represents the position of amino acid residue of EngA protein sequence in the alignment. The numbers at the top of the alignment are the positions of the multiple sequence alignment. Color codes for amino acid residues at a given position are as follows: 1) red on yellow: identical residues; 2) black on green: block of similar residues; 3) blue on cyan: conserved residues; 4) green on white: residues weakly similar to consensus residue; 5) black on white: non-similar residues. Positions of the conserved motifs in corresponding G-domains, D1 and D2 are mentioned below the aligned sequences, as represented by black bars. Sequences in the box represent switch regions in each of the two G-domains.
(TIF)

Figure S3 **Comparative analysis of** ***engA*** **locus organizations in different mycobacterial species.** Organizations of genes in *engA* locus of different mycobacterial species were analyzed by “genome region comparison” tool of CMR database (http://cmr.jcvi.org). Analysis of *engA* locus in different mycobacterial species indicates a conserved occurrence of genes preceding *engA*, which encode cytidylate kinase (*cmk*), ribosomal large subunit pseudouridine synthase B (*rluB*), segregation and condensation protein B (*scpB*), and segregation and condensation protein A (*scpA*), respectively. Genes are color coded based on function, as follows: dark blue: cellular processes; light blue: regulatory functions; black: hypothetical; white: conserved hypothetical; red: protein synthesis; orange: purines, pyrimidines, nucleosides, and nucleotides metabolism; grey: unclassified; and yellow: DNA metabolism.
(TIF)

Figure S4 **Alignment of MSMEG_3738 with Der protein sequence of** ***T. maritima.*** MSMEG_3738 was aligned with Der protein sequence of *T. maritima* by using AlignX program of Vector NTI software as described in materials and methods section. The number in parentheses before each sequence represents the position of amino acid residue of EngA protein sequence in the alignment. The numbers at the top of the alignment are the positions of the multiple sequence alignment. Color codes for amino acid residues at a given position are as described in figure 2.
(TIF)

Figure S5 ***In vitro*** **GTPase activity analysis of EngA^MS^_._** A) GTPase assay was performed as described in the text with (GTP+EngA) or without (GTP only) 1 µM EngA^MS^ in the reaction mixtures containing different concentrations of GTP. Shown is the bar graph plot using values of the rate of GTP hydrolysis (µM Pi released per hour) and concentrations of GTP (µM), represented on y- and x-axis respectively. B) GTPase assay was performed with or without heat-inactivated (H.I.) 1 µM EngA^MS^ in the reaction mixtures containing 100 µM GTP. Heat inactivation was performed at 95°C for 5 min. The x- and y-axes represent type of enzyme preparations and rate of Pi release (µM Pi released per hour) due to GTP hydrolysis, respectively. Each assay was performed in duplicate and the mean values ± s.d. were used to determine the GTPase activity.
(TIF)

Figure S6 **EngA^MS^ exhibits interaction with ribosome** ***in vivo.*** A) The *E. coli* BL21 (DE3) cells overexpressing EngA^MS^ were lysed in RNase-free environment by repeated freeze-thaw cycles and fractionated in apo form (lacking nucleotide) on 10–45% sucrose gradient prepared in low salt buffer (containing 30 mM NH_4_Cl), by ultra-centrifugation (using Beckman SW28 rotor). Equal fractions of 1 ml each were collected from top to bottom and A_254_ values for all the fractions were plotted in a graph which shows a characteristic profile of different ribosomal subunits. B) Immunoblots of the fractions containing 30S, 50S and 70S ribosomal subunits using anti-6×His antibody show EngA^MS^-specific signals that confirm an *in vivo* interaction of EngA^MS^ with ribosome.
(TIF)

Figure S7 **Both the G-domains of EngA^MS^ are required for binding with GDP.** Nucleotide binding was assayed by recording fluorescent intensities at 460 nm (λex 355 nm) upon incubating wild-type (WT) and point mutant derivatives (G4_D1 and G4_D2, respectively) of EngA^MS^ protein with fluorescent mant-nucleotide (mant-GDP), as described in the materials and methods section. The bar graph shows the relative binding of GDP with each mutant in comparison to WT at two time points of 10 min and 30 min. The values were obtained from two separate experiments and the mean values ± s.d. were used to compare the affinity of the respective proteins with GDP.
(TIF)

Figure S8 **Homology modeling predicts interactions of GD-1 and GD-2 with KH domain of EngA^MS^.** A) Homology model prediction of EngA^MS^ using structure of Der protein of *T. maritima* proposes interaction of C-terminal KH domain with both the G-domains. Specific amino acid residues involved in D1-KH interaction are part of G3 motif (B), whereas those participating in D2-KH interaction belong to G4 motif and are critical for GTP binding (C). The number next to each amino acid represents the position of amino acid residue in EngA protein sequence.
(TIF)

Table S1 **Sequences exhibiting significant alignments with MSMEG_3738.** Homologues of EngA^MS^ were obtained by blastp search as described in the materials and methods section. The table shows a list of top 100 organisms that contain EngA protein exhibiting close homology with EngA^MS^ and used in the Phylogenetic analysis. The accession number of each of the EngA proteins followed by corresponding protein name and the name of organism is shown.
(DOC)

Table S2 **List of bacterial strains, primers and plasmid constructs used in the study.** Table shows the complete list of bacterial strains, primers and plasmid constructs used in this study, as mentioned in the text. The underlined primer sequences represent respective restriction endonuclease recognition sites.
(DOC)
