# Correction: Functional Characterization of EngA^MS^, a P-Loop GTPase of *Mycobacterium smegmatis*

**DOI:** 10.1371/annotation/21849604-c4d6-4b12-bb10-435b6a5de88d

**Published:** 2012-08-20

**Authors:** Nisheeth Agarwal, Madhu Pareek, Preeti Thakur, Vibha Pathak

The legends for Supporting Information Figures S1, S2, and S3 were incorrectly switched. The correct legends for Supporting Information Figures S1, S2, and S3 are:

'''Figure S1.

Comparative analysis of engA locus organizations in different mycobacterial species.''' Organizations of genes in engA locus of different mycobacterial species were analyzed by genome region comparison” tool of CMR database (http://cmr.jcvi.orghttp://cmr.jcvi.org/>). [^] Analysis of engA locus in different mycobacterial species indicates a conserved occurrence of genes preceding engA, which encode cytidylate kinase (cmk), ribosomal large subunit pseudouridine synthase B (rluB), segregation and condensation protein B (scpB), and segregation and condensation protein A (scpA), respectively.

'''Figure S2.

Phylogenetic tree analysis of microbial EngA proteins.'''

An unrooted Phylogenetic tree was constructed from an alignment of MSMEG_3738 with the orthologous sequences as listed in Table 1, by using neighbor joining method [27]. The numbers in the parenthesis next to each organism represent the calculated distance values that reflect the degree of divergence between all pairs of sequences analyzed.

'''Figure S3.

Alignment of MSMEG_3738 with EngA protein sequences of other mycobacterial species.'''

Homologues of MSMEG_3738 were identified by blastp homology searches in different mycobacterial species that include M. abscessus (Mab), M. avium subsp. paratuberculosis K-10 (Map K-10), M. avium subsp. paratuberculosis S397 (Map S-397), M. gilvum (Mgi), M. intracellulare (Min), M. kansasii (Mka), M. leprae (Mle), M. marinum (Mma), M. parascrofulaceum (Mpa), M. tuberculosis EAS054 (Mtu EAS054), M. tuberculosis H37Rv (Mtu H37Rv), M. ulcerans (Mul), M. vanbaalenii (Mva), Mycobacterium Sp. JDM601and Mycobacterium Sp. MCS, and aligned using AlignX program of Vector NTI software as described in materials and methods section. The number in parentheses before each sequence represents the position of amino acid residue of EngA protein sequence in the alignment. The numbers at the top of the alignment are the positions of the multiple sequence alignment. Color codes for amino acid residues at a given position are as follows: 1) red on yellow: identical residues; 2) black on green: block of similar residues; 3) blue on cyan: conserved residues; 4) green on white: residues weakly similar to consensus residue; 5) black on white: non-similar residues. Positions of the conserved motifs in corresponding G-domains, D1 and D2 are mentioned below the aligned sequences, as represented by black bars. Sequences in the box represent switch regions in each of the two G-domains.

