# Supplementary figures and images for: Functional Characterization of EngAMS, a P-Loop GTPase of Mycobacterium smegmatis
Source: PLoS One. 2012 Apr 10;7(4):e34571. doi: 10.1371/journal.pone.0034571 (PMC3323550; doi:10.1371/journal.pone.0034571)

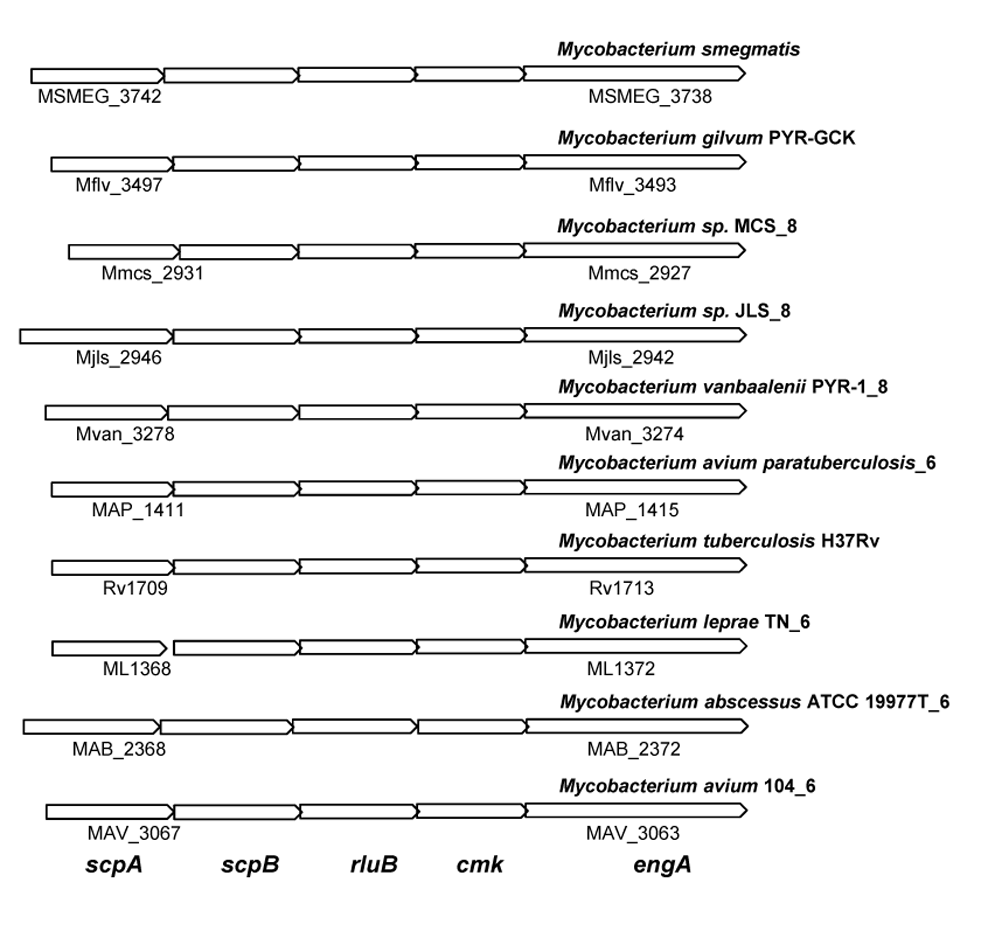

Supplement: Figure S1 — Phylogenetic tree analysis of microbial EngA proteins. An unrooted Phylogenetic tree was constructed from an alignment of MSMEG_3738 with the orthologous sequences as listed in Table 1, by using neighbor joining method [27]. The numbers in the parenthesis next to each organism represent the calculated distance values that reflect the degree of divergence between all pairs of sequences analyzed. (TIF) [file pone.0034571.s001.tif]

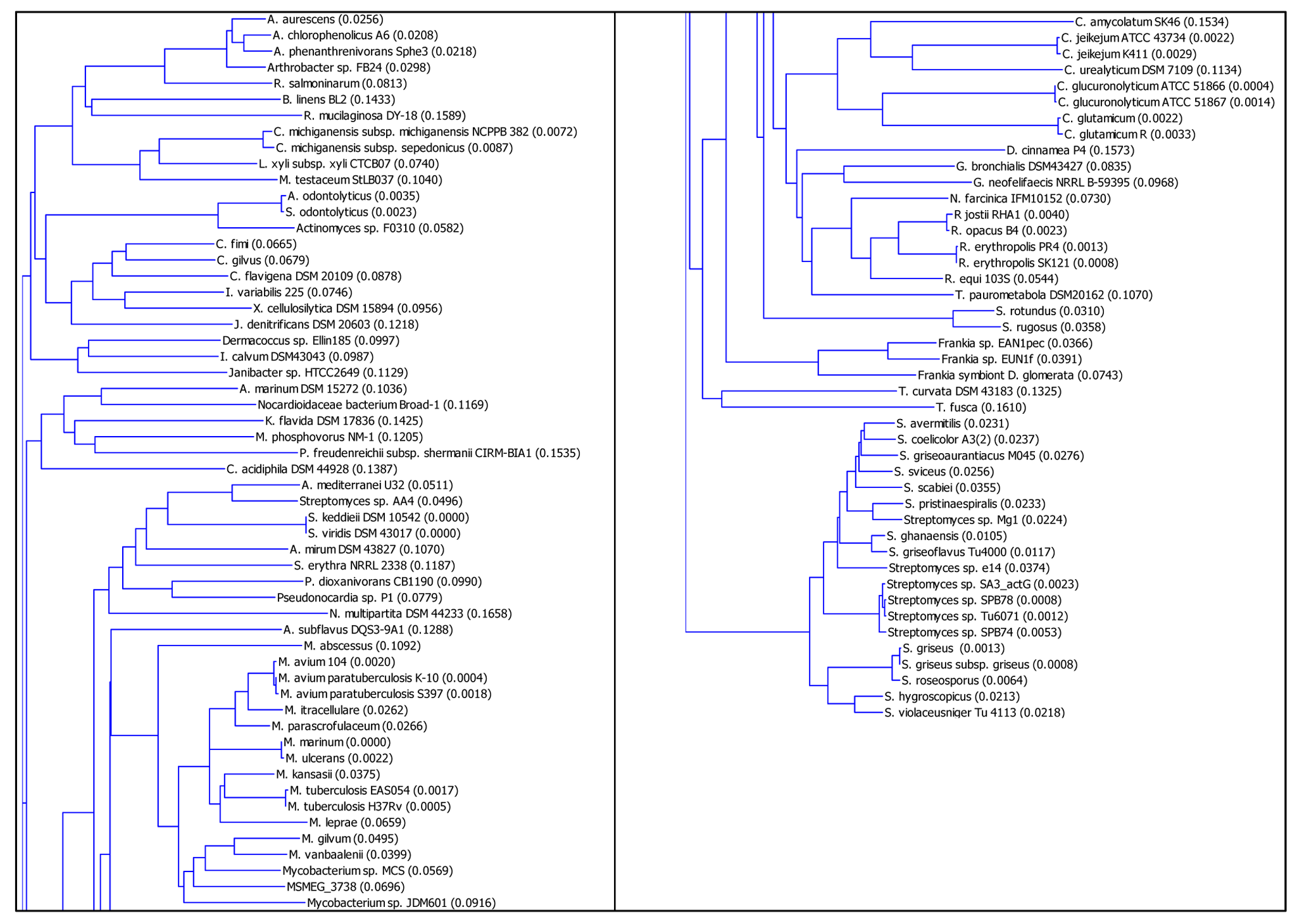

Supplement: Figure S2 — Alignment of MSMEG_3738 with EngA protein sequences of other mycobacterial species. Homologues of MSMEG_3738 were identified by blastp homology searches in different mycobacterial species that include M. abscessus (Mab), M. avium subsp. paratuberculosis K-10 (Map K-10), M. avium subsp. paratuberculosis S397 (Map S-397), M. gilvum (Mgi), M. intracellulare (Min), M. kansasii (Mka), M. leprae (Mle), M. marinum (Mma), M. parascrofulaceum (Mpa), M. tuberculosis EAS054 (Mtu EAS054), M. tuberculosis H37Rv (Mtu H37Rv), M. ulcerans (Mul), M. vanbaalenii (Mva), Mycobacterium Sp. JDM601and Mycobacterium Sp. MCS, and aligned using AlignX program of Vector NTI software as described in materials and methods section. The number in parentheses before each sequence represents the position of amino acid residue of EngA protein sequence in the alignment. The numbers at the top of the alignment are the positions of the multiple sequence alignment. Color codes for amino acid residues at a given position are as follows: 1) red on yellow: identical residues; 2) black on green: block of similar residues; 3) blue on cyan: conserved residues; 4) green on white: residues weakly similar to consensus residue; 5) black on white: non-similar residues. Positions of the conserved motifs in corresponding G-domains, D1 and D2 are mentioned below the aligned sequences, as represented by black bars. Sequences in the box represent switch regions in each of the two G-domains. (TIF) [file pone.0034571.s002.tif]

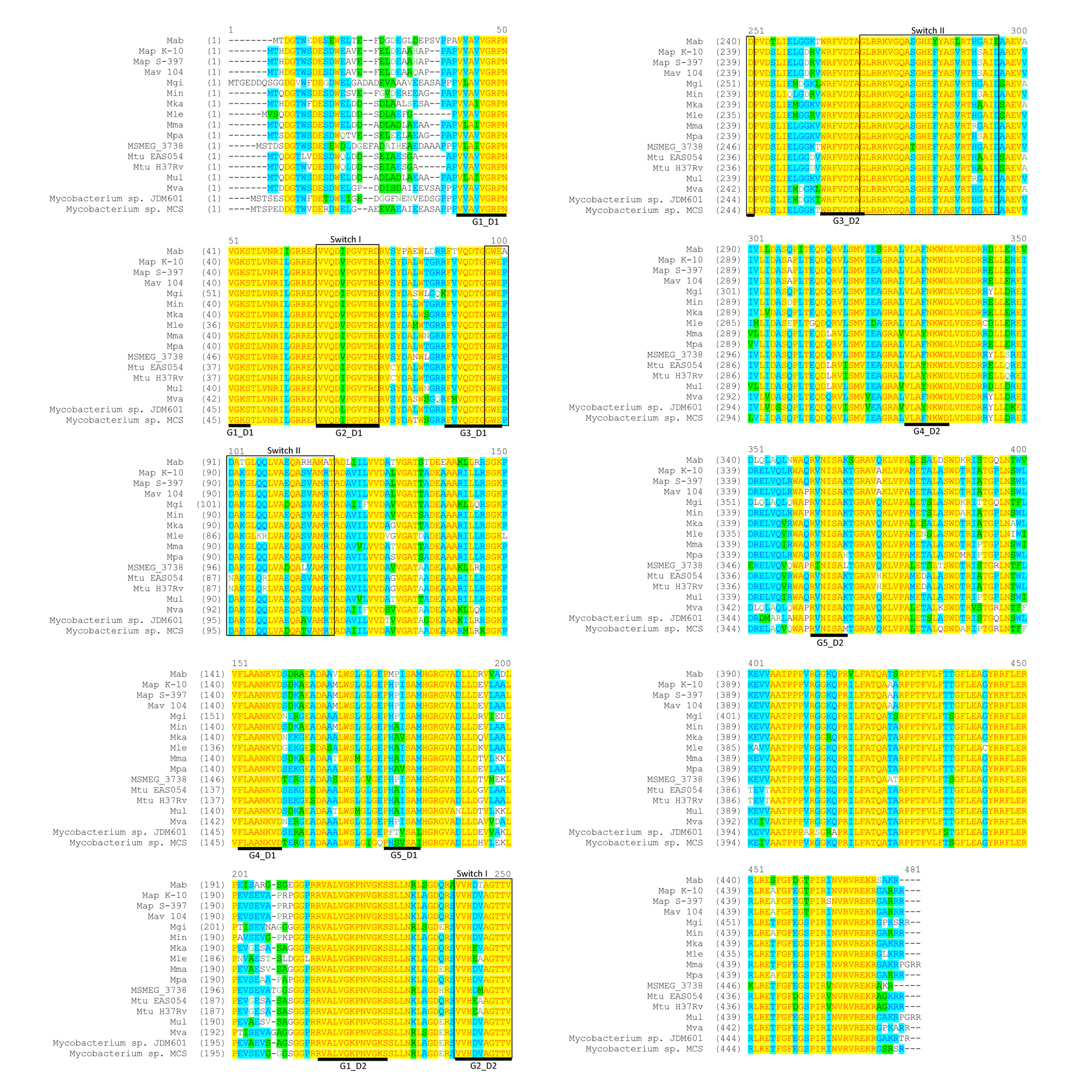

Supplement: Figure S3 — Comparative analysis of engA locus organizations in different mycobacterial species. Organizations of genes in engA locus of different mycobacterial species were analyzed by “genome region comparison” tool of CMR database (http://cmr.jcvi.org). Analysis of engA locus in different mycobacterial species indicates a conserved occurrence of genes preceding engA, which encode cytidylate kinase (cmk), ribosomal large subunit pseudouridine synthase B (rluB), segregation and condensation protein B (scpB), and segregation and condensation protein A (scpA), respectively. Genes are color coded based on function, as follows: dark blue: cellular processes; light blue: regulatory functions; black: hypothetical; white: conserved hypothetical; red: protein synthesis; orange: purines, pyrimidines, nucleosides, and nucleotides metabolism; grey: unclassified; and yellow: DNA metabolism. (TIF) [file pone.0034571.s003.tif]

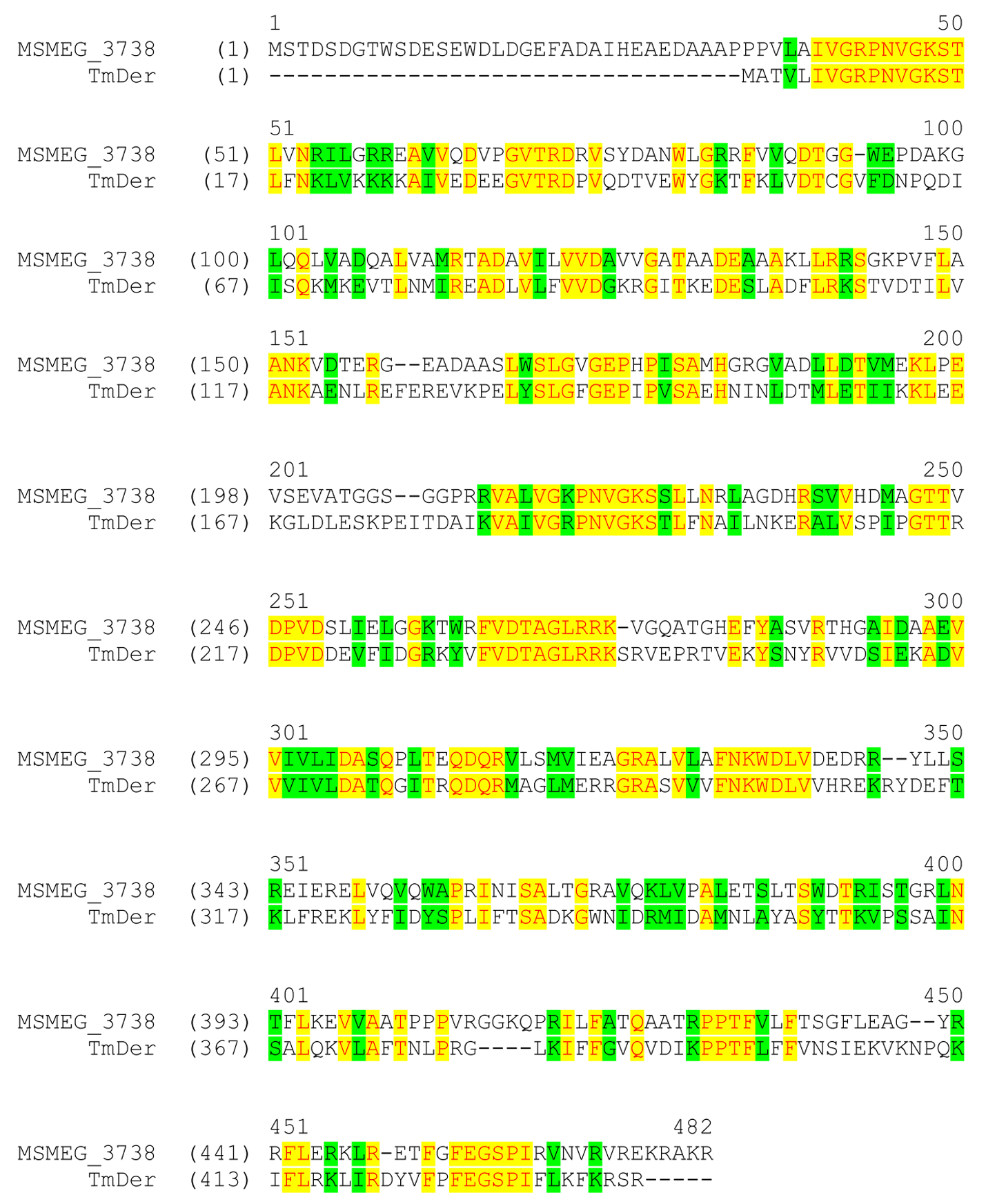

Supplement: Figure S4 — Alignment of MSMEG_3738 with Der protein sequence of T. maritima. MSMEG_3738 was aligned with Der protein sequence of T. maritima by using AlignX program of Vector NTI software as described in materials and methods section. The number in parentheses before each sequence represents the position of amino acid residue of EngA protein sequence in the alignment. The numbers at the top of the alignment are the positions of the multiple sequence alignment. Color codes for amino acid residues at a given position are as described in figure 2. (TIF) [file pone.0034571.s004.tif]

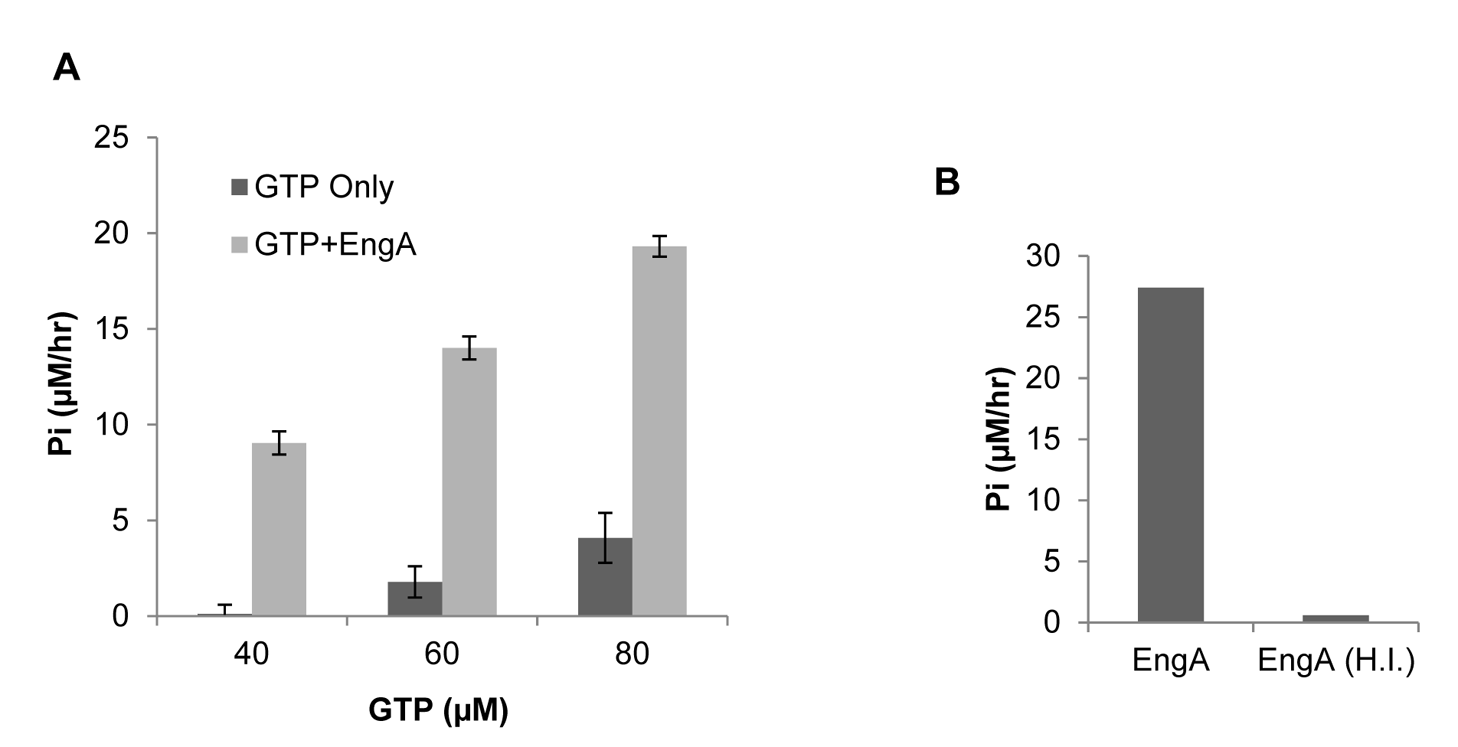

Supplement: Figure S5 — In vitro GTPase activity analysis of EngAMS. A) GTPase assay was performed as described in the text with (GTP+EngA) or without (GTP only) 1 µM EngAMS in the reaction mixtures containing different concentrations of GTP. Shown is the bar graph plot using values of the rate of GTP hydrolysis (µM Pi released per hour) and concentrations of GTP (µM), represented on y- and x-axis respectively. B) GTPase assay was performed with or without heat-inactivated (H.I.) 1 µM EngAMS in the reaction mixtures containing 100 µM GTP. Heat inactivation was performed at 95°C for 5 min. The x- and y-axes represent type of enzyme preparations and rate of Pi release (µM Pi released per hour) due to GTP hydrolysis, respectively. Each assay was performed in duplicate and the mean values ± s.d. were used to determine the GTPase activity. (TIF) [file pone.0034571.s005.tif]

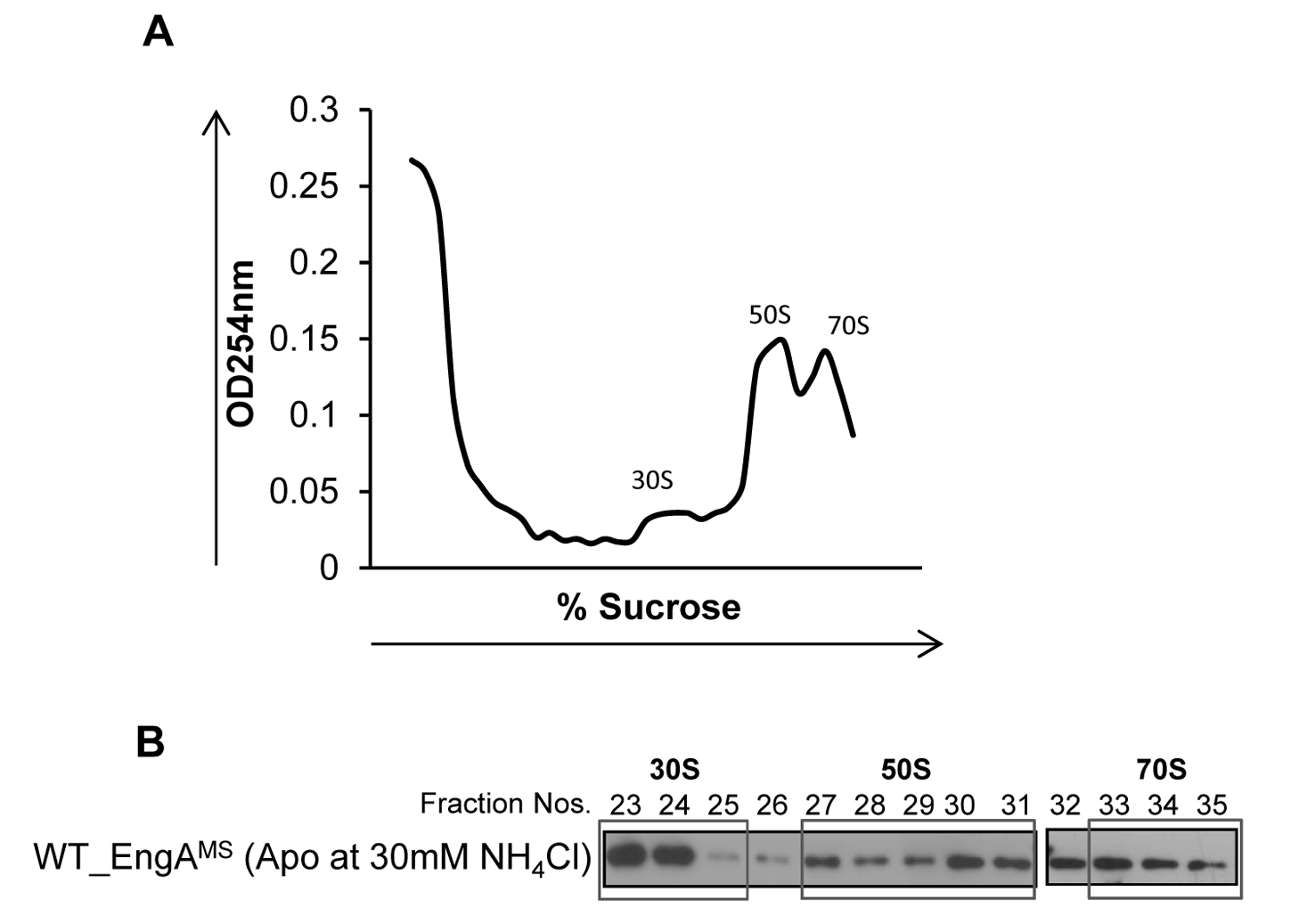

Supplement: Figure S6 — EngAMS exhibits interaction with ribosome in vivo. A) The E. coli BL21 (DE3) cells overexpressing EngAMS were lysed in RNase-free environment by repeated freeze-thaw cycles and fractionated in apo form (lacking nucleotide) on 10–45% sucrose gradient prepared in low salt buffer (containing 30 mM NH4Cl), by ultra-centrifugation (using Beckman SW28 rotor). Equal fractions of 1 ml each were collected from top to bottom and A254 values for all the fractions were plotted in a graph which shows a characteristic profile of different ribosomal subunits. B) Immunoblots of the fractions containing 30S, 50S and 70S ribosomal subunits using anti-6×His antibody show EngAMS-specific signals that confirm an in vivo interaction of EngAMS with ribosome. (TIF) [file pone.0034571.s006.tif]

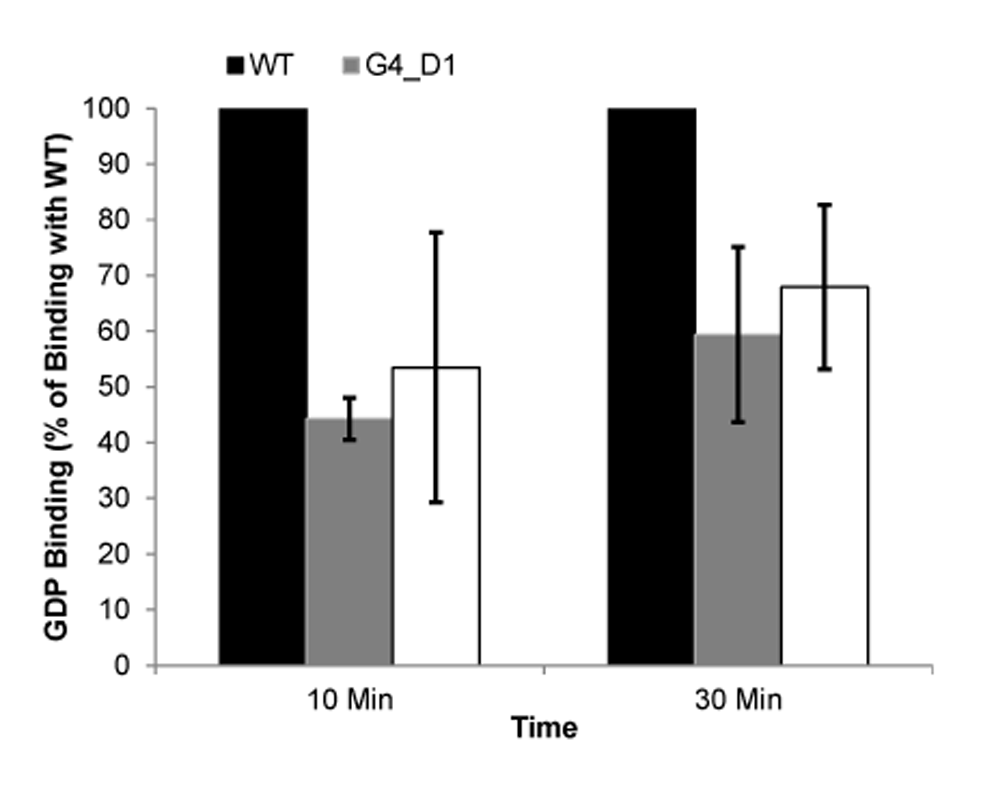

Supplement: Figure S7 — Both the G-domains of EngAMS are required for binding with GDP. Nucleotide binding was assayed by recording fluorescent intensities at 460 nm (λex 355 nm) upon incubating wild-type (WT) and point mutant derivatives (G4_D1 and G4_D2, respectively) of EngAMS protein with fluorescent mant-nucleotide (mant-GDP), as described in the materials and methods section. The bar graph shows the relative binding of GDP with each mutant in comparison to WT at two time points of 10 min and 30 min. The values were obtained from two separate experiments and the mean values ± s.d. were used to compare the affinity of the respective proteins with GDP. (TIF) [file pone.0034571.s007.tif]

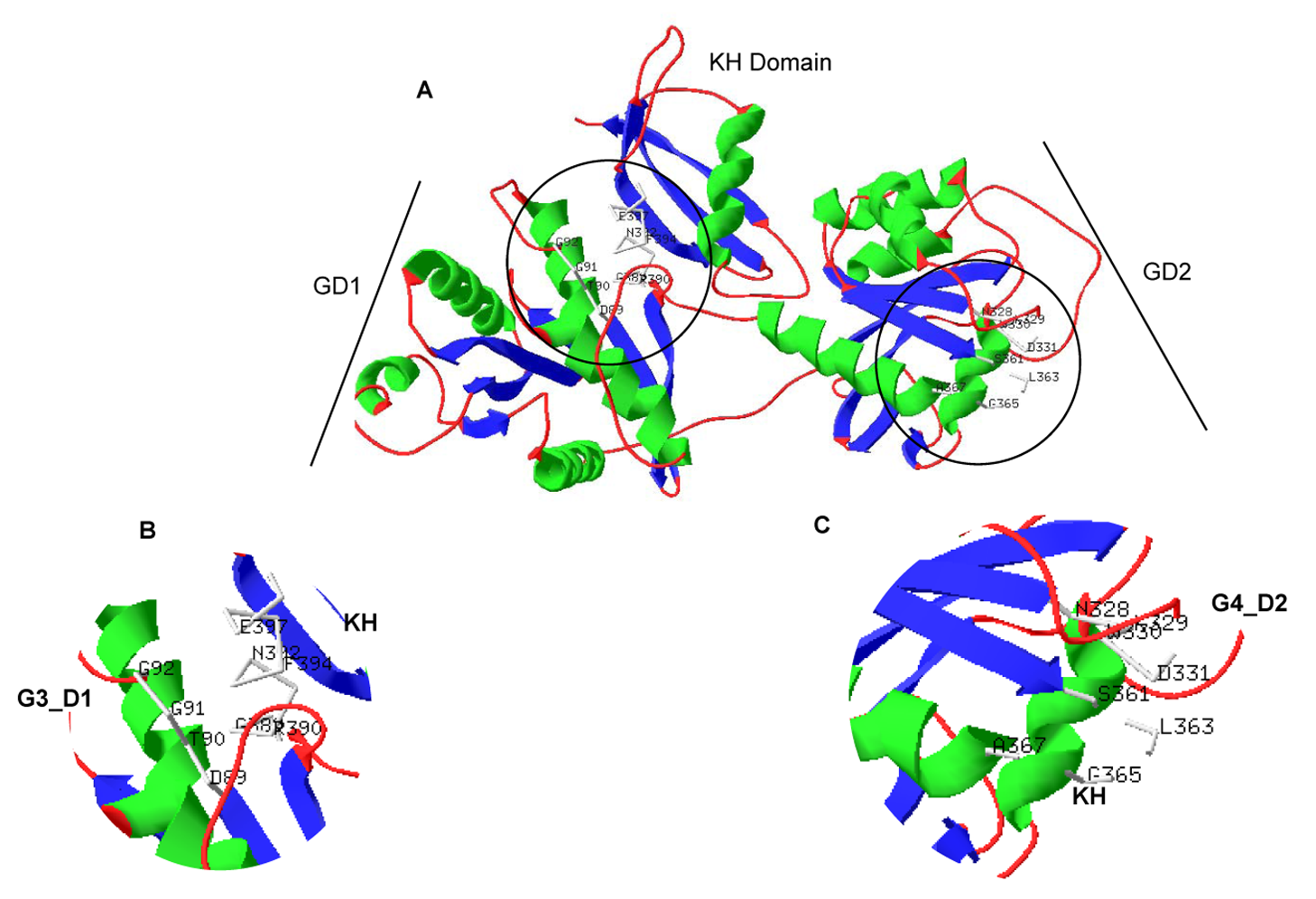

Supplement: Figure S8 — Homology modeling predicts interactions of GD-1 and GD-2 with KH domain of EngAMS. A) Homology model prediction of EngAMS using structure of Der protein of T. maritima proposes interaction of C-terminal KH domain with both the G-domains. Specific amino acid residues involved in D1-KH interaction are part of G3 motif (B), whereas those participating in D2-KH interaction belong to G4 motif and are critical for GTP binding (C). The number next to each amino acid represents the position of amino acid residue in EngA protein sequence. (TIF) [file pone.0034571.s008.tif]
