# Supplementary material for: Functional Characterization of EngAMS, a P-Loop GTPase of Mycobacterium smegmatis
Source: PLoS One. 2012 Apr 10;7(4):e34571. doi: 10.1371/journal.pone.0034571 (PMC3323550; doi:10.1371/journal.pone.0034571)
Supplement: Table S1 — Sequences exhibiting significant alignments with MSMEG_3738. Homologues of EngAMS were obtained by blastp search as described in the materials and methods section. The table shows a list of top 100 organisms that contain EngA protein exhibiting close homology with EngAMS and used in the Phylogenetic analysis. The accession number of each of the EngA proteins followed by corresponding protein name and the name of organism is shown. (DOC) [file pone.0034571.s009.doc]

**Table S1: Sequences exhibiting significant alignments with MSMEG_3738.**

| Top of Form  Bottom of Form | | |
| --- | --- | --- |
| S. No. | **Accession** | **Description** |
| 1 | [YP_888037.1](http://www.ncbi.nlm.nih.gov/protein/118468050?report=genbank&log$=prottop&blast_rank=1&RID=3C9V12M001S) | GTP-binding protein EngA [*Mycobacterium smegmatis* str. MC2 155] |
| 2 | [YP_001134755.1](http://www.ncbi.nlm.nih.gov/protein/145224077?report=genbank&log$=prottop&blast_rank=2&RID=3C9V12M001S) | GTP-binding protein EngA [*Mycobacterium gilvum* PYR-GCK] |
| 3 | [YP_954078.1](http://www.ncbi.nlm.nih.gov/protein/120404249?report=genbank&log$=prottop&blast_rank=3&RID=3C9V12M001S) | GTP-binding protein EngA [*Mycobacterium vanbaalenii* PYR-1] |
| 4 | [YP_640090.1](http://www.ncbi.nlm.nih.gov/protein/108799893?report=genbank&log$=prottop&blast_rank=4&RID=3C9V12M001S) | GTP-binding protein EngA [Mycobacterium sp. MCS] |
| 5 | [ZP_04751790.1](http://www.ncbi.nlm.nih.gov/protein/240173132?report=genbank&log$=prottop&blast_rank=5&RID=3C9V12M001S) | GTP-binding protein EngA [*Mycobacterium kansasii* ATCC 12478] |
| 6 | [ZP_06847176.1](http://www.ncbi.nlm.nih.gov/protein/296164609?report=genbank&log$=prottop&blast_rank=6&RID=3C9V12M001S) | ribosome-associated GTPase EngA [*Mycobacterium parascrofulaceum* ATCC BAA-614] |
| 7 | [YP_004523119.1](http://www.ncbi.nlm.nih.gov/protein/333990505?report=genbank&log$=prottop&blast_rank=7&RID=3C9V12M001S) | GTP-binding protein EngA [Mycobacterium sp. JDM601] |
| 8 | [YP_001850834.1](http://www.ncbi.nlm.nih.gov/protein/183982543?report=genbank&log$=prottop&blast_rank=8&RID=3C9V12M001S) | GTP-binding protein, EngA [*Mycobacterium marinum*] |
| 9 | [YP_906912.1](http://www.ncbi.nlm.nih.gov/protein/118618580?report=genbank&log$=prottop&blast_rank=9&RID=3C9V12M001S) | GTP-binding protein EngA [*Mycobacterium ulcerans* Agy99] |
| 10 | [ZP_05223900.1](http://www.ncbi.nlm.nih.gov/protein/254818899?report=genbank&log$=prottop&blast_rank=10&RID=3C9V12M001S) | GTP-binding protein EngA [*Mycobacterium intracellulare* ATCC 13950] |
| 11 | [YP_882247.1](http://www.ncbi.nlm.nih.gov/protein/118462577?report=genbank&log$=prottop&blast_rank=11&RID=3C9V12M001S) | GTP-binding protein EngA [*Mycobacterium avium* 104] |
| 12 | [NP_960349.1](http://www.ncbi.nlm.nih.gov/protein/41407513?report=genbank&log$=prottop&blast_rank=12&RID=3C9V12M001S) | GTP-binding protein EngA [*Mycobacterium avium* subsp. *paratuberculosis* K-10] |
| 13 | [EGO36861.1](http://www.ncbi.nlm.nih.gov/protein/336457867?report=genbank&log$=prottop&blast_rank=13&RID=3C9V12M001S) | ribosome-associated GTPase EngA [*Mycobacterium avium* subsp. *paratuberculosis* S397] |
| 14 | [NP_216229.1](http://www.ncbi.nlm.nih.gov/protein/15608851?report=genbank&log$=prottop&blast_rank=14&RID=3C9V12M001S) | GTP-binding protein EngA [*Mycobacterium tuberculosis* H37Rv] |
| 15 | [ZP_03428525.1](http://www.ncbi.nlm.nih.gov/protein/215430606?report=genbank&log$=prottop&blast_rank=15&RID=3C9V12M001S) | GTP-binding protein EngA [*Mycobacterium tuberculosis* EAS054] |
| 16 | [NP_301980.1](http://www.ncbi.nlm.nih.gov/protein/15827717?report=genbank&log$=prottop&blast_rank=16&RID=3C9V12M001S) | GTP-binding protein EngA [*Mycobacterium leprae* TN] |
| 17 | [YP_001703107.1](http://www.ncbi.nlm.nih.gov/protein/169629458?report=genbank&log$=prottop&blast_rank=17&RID=3C9V12M001S) | GTP-binding protein EngA [*Mycobacterium abscessus* ATCC 19977] |
| 18 | [YP_003647314.1](http://www.ncbi.nlm.nih.gov/protein/296140071?report=genbank&log$=prottop&blast_rank=18&RID=3C9V12M001S) | ribosome-associated GTPase EngA [*Tsukamurella paurometabola* DSM 20162] |
| 19 | [ZP_08206432.1](http://www.ncbi.nlm.nih.gov/protein/326384755?report=genbank&log$=prottop&blast_rank=19&RID=3C9V12M001S) | GTP-binding protein Der [*Gordonia neofelifaecis* NRRL B-59395] |
| 20 | [ZP_04388645.1](http://www.ncbi.nlm.nih.gov/protein/229494892?report=genbank&log$=prottop&blast_rank=20&RID=3C9V12M001S) | ribosome-associated GTPase EngA [*Rhodococcus erythropolis* SK121] |
| 21 | [YP_002766701.1](http://www.ncbi.nlm.nih.gov/protein/226306741?report=genbank&log$=prottop&blast_rank=21&RID=3C9V12M001S) | GTP-binding protein EngA [*Rhodococcus erythropolis* PR4] |
| 22 | [YP_004007180.1](http://www.ncbi.nlm.nih.gov/protein/312139844?report=genbank&log$=prottop&blast_rank=22&RID=3C9V12M001S) | ribosome-associated GTPase EngA [*Rhodococcus equi* ATCC 33707] |
| 23 | [YP_118223.1](http://www.ncbi.nlm.nih.gov/protein/54023981?report=genbank&log$=prottop&blast_rank=23&RID=3C9V12M001S) | GTP-binding protein EngA [*Nocardia farcinica* IFM 10152] |
| 24 | [YP_004492788.1](http://www.ncbi.nlm.nih.gov/protein/333919207?report=genbank&log$=prottop&blast_rank=24&RID=3C9V12M001S) | GTP-binding protein engA [*Amycolicicoccus subflavus* DQS3-9A1] |
| 25 | [ZP_07280340.1](http://www.ncbi.nlm.nih.gov/protein/302527998?report=genbank&log$=prottop&blast_rank=25&RID=3C9V12M001S) | ribosome-associated GTPase EngA [Streptomyces sp. AA4] |
| 26 | [YP_003768140.1](http://www.ncbi.nlm.nih.gov/protein/300787849?report=genbank&log$=prottop&blast_rank=26&RID=3C9V12M001S) | GTP-binding protein EngA [*Amycolatopsis medite*rranei U32] |
| 27 | [YP_003099411.1](http://www.ncbi.nlm.nih.gov/protein/256375751?report=genbank&log$=prottop&blast_rank=27&RID=3C9V12M001S) | GTP-binding protein EngA [*Actinosynnema mirum* DSM 43827] |
| 28 | [YP_003273922.1](http://www.ncbi.nlm.nih.gov/protein/262202714?report=genbank&log$=prottop&blast_rank=28&RID=3C9V12M001S) | ribosome-associated GTPase EngA [*Gordonia bronchialis* DSM 43247] |
| 29 | [ZP_08121313.1](http://www.ncbi.nlm.nih.gov/protein/325000201?report=genbank&log$=prottop&blast_rank=29&RID=3C9V12M001S) | GTP-binding protein Der [Pseudonocardia sp. P1] |
| 30 | [YP_002777849.1](http://www.ncbi.nlm.nih.gov/protein/226360071?report=genbank&log$=prottop&blast_rank=30&RID=3C9V12M001S) | GTP-binding protein EngA [*Rhodococcus opacus* B4] |
| 31 | [YP_700912.1](http://www.ncbi.nlm.nih.gov/protein/111017940?report=genbank&log$=prottop&blast_rank=31&RID=3C9V12M001S) | GTP-binding protein EngA [*Rhodococcus jostii* RHA1] |
| 32 | [YP_003133350.1](http://www.ncbi.nlm.nih.gov/protein/257055518?report=genbank&log$=prottop&blast_rank=32&RID=3C9V12M001S) | GTP-binding protein EngA [*Saccharomonospora viridis* DSM 43017] |
| 33 | [YP_003314741.1](http://www.ncbi.nlm.nih.gov/protein/269795286?report=genbank&log$=prottop&blast_rank=33&RID=3C9V12M001S) | hypothetical protein Sked_19830 [*Sanguibacter keddieii* DSM 10542] |
| 34 | [YP_004600740.1](http://www.ncbi.nlm.nih.gov/protein/336320772?report=genbank&log$=prottop&blast_rank=34&RID=3C9V12M001S) | ribosome-associated GTPase EngA [*Cellvibrio gilvus* ATCC 13127] |
| 35 | [YP_001107397.1](http://www.ncbi.nlm.nih.gov/protein/134101736?report=genbank&log$=prottop&blast_rank=35&RID=3C9V12M001S) | GTP-binding protein EngA [*Saccharopolyspora erythraea* NRRL 2338] |
| 36 | [ZP_07704208.1](http://www.ncbi.nlm.nih.gov/protein/309810373?report=genbank&log$=prottop&blast_rank=36&RID=3C9V12M001S) | ribosome biogenesis GTPase Der [*Dermacoccus* sp. Ellin185] |
| 37 | [YP_003381098.1](http://www.ncbi.nlm.nih.gov/protein/284031167?report=genbank&log$=prottop&blast_rank=37&RID=3C9V12M001S) | small GTP-binding protein [*Kribbella flavida* DSM 17836] |
| 38 | [YP_004542159.1](http://www.ncbi.nlm.nih.gov/protein/334337007?report=genbank&log$=prottop&blast_rank=38&RID=3C9V12M001S) | GTP-binding protein engA [*Isoptericola variabilis* 225] |
| 39 | [ZP_07964633.1](http://www.ncbi.nlm.nih.gov/protein/317506861?report=genbank&log$=prottop&blast_rank=39&RID=3C9V12M001S) | ribosome-associated GTPase EngA [*Segniliparus rugosus* ATCC BAA-974] |
| 40 | [YP_003637018.1](http://www.ncbi.nlm.nih.gov/protein/296129768?report=genbank&log$=prottop&blast_rank=40&RID=3C9V12M001S) | ribosome-associated GTPase EngA [*Cellulomonas flavigena* DSM 20109] |
| 41 | [ZP_08023269.1](http://www.ncbi.nlm.nih.gov/protein/319949180?report=genbank&log$=prottop&blast_rank=41&RID=3C9V12M001S) | GTP-binding protein Der [*Dietzia cinnamea* P4] |
| 42 | [YP_003113809.1](http://www.ncbi.nlm.nih.gov/protein/256392245?report=genbank&log$=prottop&blast_rank=42&RID=3C9V12M001S) | small GTP-binding protein [*Catenulispora acidiphila* DSM 44928] |
| 43 | [YP_004453401.1](http://www.ncbi.nlm.nih.gov/protein/332670393?report=genbank&log$=prottop&blast_rank=43&RID=3C9V12M001S) | ribosome-associated GTPase EngA [*Cellulomonas fimi* ATCC 484] |
| 44 | [ZP_06412817.1](http://www.ncbi.nlm.nih.gov/protein/288918466?report=genbank&log$=prottop&blast_rank=44&RID=3C9V12M001S) | small GTP-binding protein [Frankia sp. EUN1f] |
| 45 | [ZP_00995224.1](http://www.ncbi.nlm.nih.gov/protein/84496370?report=genbank&log$=prottop&blast_rank=45&RID=3C9V12M001S) | GTP-binding protein EngA [Janibacter sp. HTCC2649] |
| 46 | [ZP_07298862.1](http://www.ncbi.nlm.nih.gov/protein/302546520?report=genbank&log$=prottop&blast_rank=46&RID=3C9V12M001S) | ribosome-associated GTPase EngA [*Streptomyces hygroscopicus* ATCC 53653] |
| 47 | [YP_061644.1](http://www.ncbi.nlm.nih.gov/protein/50954356?report=genbank&log$=prottop&blast_rank=47&RID=3C9V12M001S) | GTP-binding protein EngA [*Leifsonia xyli* subsp. *xyli* str. CTCB07] |
| 48 | [YP_003203330.1](http://www.ncbi.nlm.nih.gov/protein/258654174?report=genbank&log$=prottop&blast_rank=48&RID=3C9V12M001S) | small GTP-binding protein [*Nakamurella multipartita* DSM 44233] |
| 49 | [YP_004333826.1](http://www.ncbi.nlm.nih.gov/protein/331697587?report=genbank&log$=prottop&blast_rank=49&RID=3C9V12M001S) | GTP-binding protein engA [*Pseudonocardia dioxanivorans* CB1190] |
| 50 | [YP_003161231.1](http://www.ncbi.nlm.nih.gov/protein/256832504?report=genbank&log$=prottop&blast_rank=50&RID=3C9V12M001S) | small GTP-binding protein [*Jonesia denitrificans* DSM 20603] |
| 51 | [YP_831032.1](http://www.ncbi.nlm.nih.gov/protein/116670099?report=genbank&log$=prottop&blast_rank=51&RID=3C9V12M001S) | GTP-binding protein EngA [Arthrobacter sp. FB24] |
| 52 | [YP_001222716.1](http://www.ncbi.nlm.nih.gov/protein/148273155?report=genbank&log$=prottop&blast_rank=52&RID=3C9V12M001S) | GTP-binding protein EngA [*Clavibacter michiganensis* subsp. *michiganensis* NCPPB 382] |
| 53 | [ZP_05001171.1](http://www.ncbi.nlm.nih.gov/protein/254385852?report=genbank&log$=prottop&blast_rank=53&RID=3C9V12M001S) | GTP-binding protein engA [Streptomyces sp. Mg1] |
| 54 | [ZP_07608097.1](http://www.ncbi.nlm.nih.gov/protein/307328928?report=genbank&log$=prottop&blast_rank=54&RID=3C9V12M001S) | ribosome-associated GTPase EngA [*Streptomyces violaceusniger* Tu 4113] |
| 55 | [YP_001709996.1](http://www.ncbi.nlm.nih.gov/protein/170781664?report=genbank&log$=prottop&blast_rank=55&RID=3C9V12M001S) | GTP-binding protein EngA [*Clavibacter michiganensis* subsp. *sepedonicus*] |
| 56 | [YP_003326153.1](http://www.ncbi.nlm.nih.gov/protein/269956364?report=genbank&log$=prottop&blast_rank=56&RID=3C9V12M001S) | cytidylate kinase [*Xylanimonas cellulosilytica* DSM 15894] |
| 57 | [ZP_07716229.1](http://www.ncbi.nlm.nih.gov/protein/311742420?report=genbank&log$=prottop&blast_rank=57&RID=3C9V12M001S) | ribosome-associated GTPase EngA [*Aeromicrobium marinum* DSM 15272] |
| 58 | [YP_004223101.1](http://www.ncbi.nlm.nih.gov/protein/323356705?report=genbank&log$=prottop&blast_rank=58&RID=3C9V12M001S) | GTPase [*Microbacterium testaceum* StLB037] |
| 59 | [ZP_06707149.1](http://www.ncbi.nlm.nih.gov/protein/294628589?report=genbank&log$=prottop&blast_rank=59&RID=3C9V12M001S) | ribosome-associated GTPase EngA [Streptomyces sp. e14] |
| 60 | [ZP_07980124.1](http://www.ncbi.nlm.nih.gov/protein/318061403?report=genbank&log$=prottop&blast_rank=60&RID=3C9V12M001S) | GTP-binding protein Der [Streptomyces sp. SA3_actG] |
| 61 | [ZP_06916066.1](http://www.ncbi.nlm.nih.gov/protein/297198669?report=genbank&log$=prottop&blast_rank=61&RID=3C9V12M001S) | ribosome-associated GTPase EngA [*Streptomyces sviceus* ATCC 29083] |
| 62 | [YP_004240823.1](http://www.ncbi.nlm.nih.gov/protein/325962917?report=genbank&log$=prottop&blast_rank=62&RID=3C9V12M001S) | GTP-binding protein Era [*Arthrobacter phenanthrenivorans* Sphe3] |
| 63 | [EGE45377.1](http://www.ncbi.nlm.nih.gov/protein/326660531?report=genbank&log$=prottop&blast_rank=63&RID=3C9V12M001S) | ribosome-associated GTPase EngA [*Streptomyces griseus* XylebKG-1] |
| 64 | [YP_001827253.1](http://www.ncbi.nlm.nih.gov/protein/182439534?report=genbank&log$=prottop&blast_rank=64&RID=3C9V12M001S) | GTP-binding protein EngA [*Streptomyces griseus* subsp. *griseus* NBRC 13350] |
| 65 | [ZP_04692163.1](http://www.ncbi.nlm.nih.gov/protein/239940226?report=genbank&log$=prottop&blast_rank=65&RID=3C9V12M001S) | GTP-binding protein EngA [*Streptomyces roseosporus* NRRL 15998] |
| 66 | [ZP_08456154.1](http://www.ncbi.nlm.nih.gov/protein/333028090?report=genbank&log$=prottop&blast_rank=66&RID=3C9V12M001S) | putative GTP-binding protein EngA [Streptomyces sp. Tu6071] |
| 67 | [NP_827700.1](http://www.ncbi.nlm.nih.gov/protein/29833066?report=genbank&log$=prottop&blast_rank=67&RID=3C9V12M001S) | GTP-binding protein EngA [*Streptomyces avermitilis* MA-4680] |
| 68 | [YP_001625727.1](http://www.ncbi.nlm.nih.gov/protein/163841322?report=genbank&log$=prottop&blast_rank=68&RID=3C9V12M001S) | GTP-binding protein EngA [*Renibacterium salmoninarum* ATCC 33209] |
| 69 | [ZP_04689118.1](http://www.ncbi.nlm.nih.gov/protein/239932165?report=genbank&log$=prottop&blast_rank=69&RID=3C9V12M001S) | GTP-binding protein EngA [*Streptomyces ghanaensis* ATCC 14672] |
| 70 | [YP_004099072.1](http://www.ncbi.nlm.nih.gov/protein/317124960?report=genbank&log$=prottop&blast_rank=70&RID=3C9V12M001S) | ribosome-associated GTPase EngA [*Intrasporangium calvum* DSM 43043] |
| 71 | [ZP_07270575.1](http://www.ncbi.nlm.nih.gov/protein/302518233?report=genbank&log$=prottop&blast_rank=71&RID=3C9V12M001S) | ribosome-associated GTPase EngA [Streptomyces sp. SPB78] |
| 72 | [ZP_06609686.1](http://www.ncbi.nlm.nih.gov/protein/293192791?report=genbank&log$=prottop&blast_rank=72&RID=3C9V12M001S) | ribosome-associated GTPase EngA [*Actinomyces odontolyticus* F0309] |
| 73 | [ZP_08288578.1](http://www.ncbi.nlm.nih.gov/protein/329939204?report=genbank&log$=prottop&blast_rank=73&RID=3C9V12M001S) | GTP-binding protein EngA [*Streptomyces griseoaurantiacus* M045] |
| 74 | [ZP_07313789.1](http://www.ncbi.nlm.nih.gov/protein/302561447?report=genbank&log$=prottop&blast_rank=74&RID=3C9V12M001S) | ribosome-associated GTPase EngA [*Streptomyces griseoflavus* Tu4000] |
| 75 | [YP_003492726.1](http://www.ncbi.nlm.nih.gov/protein/290961544?report=genbank&log$=prottop&blast_rank=75&RID=3C9V12M001S) | GTPase [*Streptomyces scabiei* 87.22] |
| 76 | [YP_947439.1](http://www.ncbi.nlm.nih.gov/protein/119964121?report=genbank&log$=prottop&blast_rank=76&RID=3C9V12M001S) | GTP-binding protein EngA [*Arthrobacter aurescens* TC1] |
| 77 | [ZP_07879272.1](http://www.ncbi.nlm.nih.gov/protein/315604206?report=genbank&log$=prottop&blast_rank=77&RID=3C9V12M001S) | ribosome-associated GTPase EngA [Actinomyces sp. oral taxon 180 str. F0310] |
| 78 | [YP_004583164.1](http://www.ncbi.nlm.nih.gov/protein/336177789?report=genbank&log$=prottop&blast_rank=78&RID=3C9V12M001S) | GTP-binding protein engA [Frankia symbiont of Datisca glomerata] |
| 79 | [YP_289271.1](http://www.ncbi.nlm.nih.gov/protein/72161614?report=genbank&log$=prottop&blast_rank=79&RID=3C9V12M001S) | GTP-binding protein EngA [*Thermobifida fusca* YX] |
| 80 | [ZP_06912622.1](http://www.ncbi.nlm.nih.gov/protein/297195224?report=genbank&log$=prottop&blast_rank=80&RID=3C9V12M001S) | GTP-binding protein engA [*Streptomyces pristinaespiralis* ATCC 25486] |
| 81 | [YP_003658600.1](http://www.ncbi.nlm.nih.gov/protein/296393716?report=genbank&log$=prottop&blast_rank=81&RID=3C9V12M001S) | ribosome-associated GTPase EngA [*Segniliparus rotundus* DSM 44985] |
| 82 | [ZP_02044454.1](http://www.ncbi.nlm.nih.gov/protein/154508812?report=genbank&log$=prottop&blast_rank=82&RID=3C9V12M001S) | hypothetical protein ACTODO_01321 [*Actinomyces odontolyticus* ATCC 17982] |
| 83 | [NP_600646.1](http://www.ncbi.nlm.nih.gov/protein/19552644?report=genbank&log$=prottop&blast_rank=83&RID=3C9V12M001S) | GTP-binding protein EngA [*Corynebacterium glutamicum* ATCC 13032] |
| 84 | [ZP_05913585.1](http://www.ncbi.nlm.nih.gov/protein/260905263?report=genbank&log$=prottop&blast_rank=84&RID=3C9V12M001S) | GTP-binding protein EngA [*Brevibacterium linens* BL2] |
| 85 | [YP_001138383.1](http://www.ncbi.nlm.nih.gov/protein/145295562?report=genbank&log$=prottop&blast_rank=85&RID=3C9V12M001S) | GTP-binding protein EngA [*Corynebacterium glutamicum* R] |
| 86 | [NP_626030.1](http://www.ncbi.nlm.nih.gov/protein/21220251?report=genbank&log$=prottop&blast_rank=86&RID=3C9V12M001S) | GTP-binding protein EngA [*Streptomyces coelicolor* A3(2)] |
| 87 | [YP_003300409.1](http://www.ncbi.nlm.nih.gov/protein/269127039?report=genbank&log$=prottop&blast_rank=87&RID=3C9V12M001S) | small GTP-binding protein [*Thermomonospora curvata* DSM 43183] |
| 88 | [YP_002487615.1](http://www.ncbi.nlm.nih.gov/protein/220912306?report=genbank&log$=prottop&blast_rank=88&RID=3C9V12M001S) | GTP-binding protein EngA [*Arthrobacter chlorophenolicus* A6] |
| 89 | [YP_001509323.1](http://www.ncbi.nlm.nih.gov/protein/158316815?report=genbank&log$=prottop&blast_rank=89&RID=3C9V12M001S) | GTP-binding protein EngA [Frankia sp. EAN1pec] |
| 90 | [ZP_03394195.1](http://www.ncbi.nlm.nih.gov/protein/213966005?report=genbank&log$=prottop&blast_rank=90&RID=3C9V12M001S) | bifunctional cytidylate kinase/GTP-binding protein [*Corynebacterium amycolatum* SK46] |
| 91 | [YP_003688078.1](http://www.ncbi.nlm.nih.gov/protein/297626315?report=genbank&log$=prottop&blast_rank=91&RID=3C9V12M001S) | GTP binding protein [*Propionibacterium freudenreichii* subsp. *shermanii* CIRM-BIA1] |
| 92 | [YP_001800481.1](http://www.ncbi.nlm.nih.gov/protein/172040767?report=genbank&log$=prottop&blast_rank=92&RID=3C9V12M001S) | bifunctional cytidylate kinase/GTP-binding protein [*Corynebacterium urealyticum* DSM 7109] |
| 93 | [YP_250660.1](http://www.ncbi.nlm.nih.gov/protein/68535955?report=genbank&log$=prottop&blast_rank=93&RID=3C9V12M001S) | bifunctional cytidylate kinase/GTP-binding protein [*Corynebacterium jeikeium* K411] |
| 94 | [ZP_05846570.1](http://www.ncbi.nlm.nih.gov/protein/260578662?report=genbank&log$=prottop&blast_rank=94&RID=3C9V12M001S) | ribosome-associated GTPase EngA [*Corynebacterium jeikeium* ATCC 43734] |
| 95 | [ZP_08197579.1](http://www.ncbi.nlm.nih.gov/protein/326331287?report=genbank&log$=prottop&blast_rank=95&RID=3C9V12M001S) | ribosome-associated GTPase EngA [Nocardioidaceae bacterium Broad-1] |
| 96 | [YP_004573024.1](http://www.ncbi.nlm.nih.gov/protein/336118255?report=genbank&log$=prottop&blast_rank=96&RID=3C9V12M001S) | GTP-binding protein EngA [*Microlunatus phosphovorus* NM-1] |
| 97 | [YP_003363045.1](http://www.ncbi.nlm.nih.gov/protein/283458421?report=genbank&log$=prottop&blast_rank=97&RID=3C9V12M001S) | putative GTPase [*Rothia mucilaginosa* DY-18] |
| 98 | [ZP_03919313.1](http://www.ncbi.nlm.nih.gov/protein/227488997?report=genbank&log$=prottop&blast_rank=98&RID=3C9V12M001S) | GTP-binding protein EngA [*Corynebacterium glucuronolyticum* ATCC 51867] |
| 99 | [ZP_03972059.1](http://www.ncbi.nlm.nih.gov/protein/227542010?report=genbank&log$=prottop&blast_rank=99&RID=3C9V12M001S) | GTP-binding protein EngA [*Corynebacterium glucuronolyticum* ATCC 51866] |
| 100 | [ZP_06826635.1](http://www.ncbi.nlm.nih.gov/protein/295839702?report=genbank&log$=prottop&blast_rank=100&RID=3C9V12M001S) | ribosome-associated GTPase EngA [Streptomyces sp. SPB74] |

Homologues of EngA^MS^ were obtained by blastp search as described in the materials and methods section. The table shows a list of top 100 organisms that contain EngA protein exhibiting close homology with EngA^MS^ and used in the Phylogenetic analysis. The accession number of each of the EngA proteins followed by corresponding protein name and the name of organism is shown.
