# Supplementary material for: Functional Characterization of EngAMS, a P-Loop GTPase of Mycobacterium smegmatis
Source: PLoS One. 2012 Apr 10;7(4):e34571. doi: 10.1371/journal.pone.0034571 (PMC3323550; doi:10.1371/journal.pone.0034571)
Supplement: Table S2 — List of bacterial strains, primers and plasmid constructs used in the study. Table shows the complete list of bacterial strains, primers and plasmid constructs used in this study, as mentioned in the text. The underlined primer sequences represent respective restriction endonuclease recognition sites. (DOC) [file pone.0034571.s010.doc]

**Table S2: List of bacterial strains, primers and plasmid constructs used in the study.**

| **A. Bacterial Strains:** | | | |
| --- | --- | --- | --- |
| **S. No.** | **Name** | **Genotype/Description** | **Source** |
| 1. | *E. coli* DH5α | *F’*/*endA1 hsdR17* [r_k_^-^m_k_^+^] *glnV44 thi-1 recA1gyrA* [Nal^r^] *relA1* Δ[lac*IZYA-argF*]*U169* *deoR* [Φ80*dlac*Δ(*lacZ*)*M15*] | Stratagene |
| 2. | *E. coli* BL21 (DE3) | *F*- *ompT hsdS_B_* [r_B_^-^m_B_^-^] *gal dcm* [DE3] | Novagen |
| 3. | *M. smegmatis* MC^2^155 | Avirulent, fast growing saprophytic strain of mycobacterium | A kind gift from Dr. William Bishai, Johns Hopkins University School of Medicine, Baltimore, USA |
| **B. Primers:** | | | |
| **S. No.** | **Name** | **Sequence** | |
| 1. | MS3738_F | 5'- GGCCATATGAGCACCGATTCCGACG-3' | |
| 2. | MS3738_R | 5'- GGCAAGCTTCTAACGCTTCGCTCGTTTTT-3' | |
| 3. | D1_F | 5'-CAACAAGGTTAACACCGAGCG-3' | |
| 4. | D1_R | 5'-CGCTCGGTGTTAACCTTGTTG-3' | |
| 5. | D2_F | 5'-CAACAAATGGAACCTGGTCGAC-3' | |
| 6. | D2_R | 5'-GTCGACCAGGTTCCATTTGTTG-3' | |
| 7. | Cter_F | 5'-CATCAGTGCCATATGCACGGCC-3' | |
| 8. | Cter_R | 5'-GGCCGTGCATATGGCACTGATG-3' | |
| **C. Plasmids:** | | | |
| **S. No.** | **Name** | **Description** | **Reference** |
| 1. | pET-28a | An expression vector with pBR322 plasmid origin of DNA replication and T7 promoter. | Novagen |
| 2. | pET*28-EngA^MS^* | pET28a containing wild-type *MSMEG_3738* for the expression of EngA^MS^ in *E. coli* by IPTG induction. | This study |
| 3. | pET*28- Cter-EngA^MS^* | pET28a containing N-terminal deletion mutant of *MSMEG_3738* for the expression of C-terminal domains, D2 and KH of EngA^MS^ in *E. coli* by IPTG induction. | This study |

Table shows the complete list of bacterial strains, primers and plasmid constructs used in this study, as mentioned in the text. The underlined primer sequences represent respective restriction endonuclease recognition sites.
